# Effects of 4 Testing Arena Sizes and 11 Types of Embryo Media on Sensorimotor Behaviors in Wild-Type and *chd7* Mutant Zebrafish Larvae

**DOI:** 10.1089/zeb.2023.0052

**Published:** 2024-02-14

**Authors:** Dana R. Hodorovich, Tiara Fryer Harris, Derek F. Burton, Katie M. Neese, Rachael A. Bieler, Vimal Chudasama, Kurt C. Marsden

**Affiliations:** Department of Biological Sciences, North Carolina State University, Raleigh, North Carolina, USA.

**Keywords:** zebrafish, behavior, acoustic startle, embryo media, arena size, *chd7*

## Abstract

The larval zebrafish is a highly versatile model across research disciplines, and the expanding use of behavioral analysis has contributed to many advances in neuropsychiatric, developmental, and toxicological studies, often through large-scale chemical and genetic screens. In the absence of standardized approaches to larval zebrafish behavior analysis, however, it is critical to understand the impact on behavior of experimental variables such as the size of testing arenas and the choice of embryo medium. Using a custom-built, modular high-throughput testing system, we examined the effects of 4 testing arena sizes and 11 types of embryo media on conserved sensorimotor behaviors in zebrafish larvae. Our data show that testing arena size impacts acoustic startle sensitivity and kinematics, as well as spontaneous locomotion and thigmotaxis, with fish tested in larger arenas displaying reduced startle sensitivity and increased locomotion. We also find that embryo media can dramatically affect startle sensitivity, kinematics, habituation, and prepulse inhibition, as well as spontaneous swimming, turning, and overall activity. Common medium components such as methylene blue and high calcium concentration consistently reduced startle sensitivity and locomotion. To further address how the choice of embryo medium can impact phenotype expression in zebrafish models of disease, we reared *chd7* mutant larvae, a model of CHARGE syndrome with previously characterized morphological and behavioral phenotypes, in five different types of media and observed impacts on all phenotypes. By defining the effects of these key extrinsic factors on larval zebrafish behavior, these data can help researchers select the most appropriate conditions for their specific research questions, particularly for genetic and chemical screens.

## Introduction

The teleost *Danio rerio* or zebrafish has become a powerful model in biological and biomedical research. The larval zebrafish is an accessible and versatile model across multiple research fields, including neuroscience, genetics, developmental biology, toxicology, immunology, and more.^1–7^ Advantages of the zebrafish model include external fertilization and development, transparent embryos and larvae, high fecundity, cost-effective maintenance,^8^ and defined orthologs for ∼70% of human genes.^9^ As use of the larval zebrafish model continues to increase, particularly with high-throughput approaches using larval zebrafish behavior as a readout of neural function, it has become more important to define how key experimental variables affect larval development and behavior.

By 5 days postfertilization (dpf), larvae reliably perform conserved auditory- and visually mediated behaviors,^10–13^ making them a powerful tool for multiple research questions in developmental neuroscience, toxicology, and chemical or genetic screening.^1,4,14,15^ For example, high-throughput screening of visual and locomotor behavior of larvae treated with various compounds is an established measure of toxicity.^6,14^

However, while standardized protocols for comparable experiments with rodents have been well established,^16^ protocols for raising and testing zebrafish larvae can vary widely, and experimental variables such as testing arena size and embryo medium have been reported to impact larval zebrafish behavior.^17,18^ The influence of media on phenotypes is especially critical when screening mutant lines, where media may have a genotype-specific effect that could mask potential differences. Therefore, to ensure the reliability and reproducibility of findings from laboratories around the world, it is necessary to define the effects that testing arena size and embryo medium type have on a range of larval zebrafish behaviors.

In this study, we assayed multiple behaviors, including the acoustic startle response, short-term habituation, prepulse inhibition (PPI), and spontaneous locomotion, as well as kinematic performance in larvae tested in 4 arena sizes and raised in 11 different media. In addition, we tested an established zebrafish model of CHARGE Syndrome^19^ (*chd7^ncu101/^*) with defined morphological and behavioral phenotypes in different media to assess the impact on phenotype expression. Our results show arena- and medium-dependent differences affecting multiple sensorimotor behaviors.

Increasing testing arena diameter significantly decreased startle frequency and increased thigmotaxis. In addition, media with additives, including methylene blue, higher calcium concentrations, and sodium bicarbonate, exerted significant and bidirectional changes in both conserved auditory and general locomotor behaviors compared to control embryo media (1 × E3). Finally, homozygous *chd7* mutants (*chd7^ncu101/ncu101^*) displayed the highest penetrance of morphological phenotypes and significant changes in auditory-driven behaviors when reared in media containing methylene blue. We observed high degrees of variation within the same genotype across medium types, indicating a genotype-specific influence on auditory and locomotor behaviors. Together these results demonstrate how extrinsic experimental factors impact larval zebrafish behavior as well as phenotypic penetrance in a zebrafish disease model.

## Materials and Methods

### Zebrafish husbandry and maintenance

All animal use and procedures were approved by the North Carolina State University Institutional Animal Care and Use Committee (IACUC). All testing was performed with the *Tüpfel long fin* (TLF) strain. The wild-type TLF line originated from University of Pennsylvania stocks, and the *chd7^ncu101/+^* line was maintained in the TLF strain, which originated from Zebrafish International Resource Center (ZIRC) stocks. Adult zebrafish were housed in 5 fish/L density under a 14-h light/10-h dark cycle at ∼28°C, and were fed rotifers, *Artemia* brine shrimp (Brine Shrimp Direct), and GEMMA micro 300 (Skretting).

To generate embryos for larval testing, male and female pairs were placed in mating boxes (Aquaneering) containing system water and artificial grass. One to two hours into the subsequent light cycle of the following day, embryos were collected and placed into Petri dishes containing 1 × E3 media with 0.05% methylene blue. Embryos were sorted for fertilization under a dissecting scope at ∼6 h postfertilization (hpf) and placed into 10 cm Petri dishes with *n* ≤ 60.

Clutches were evenly mixed such that each dish represented equal numbers of embryos from multiple clutches. For media experiments, 1 × E3 medium was completely removed and embryos were placed in experimental media following sorting. All embryos were reared in a temperature-controlled incubator at 29°C on a 14-h light/10-h dark cycle. Each day until testing, a 50–75% medium change was performed. For medium experiments, each medium was tested across multiple batches of embryos, and every condition was tested on a minimum of two experimental days. All experiments were performed blind to genotype, when applicable.

### Embryo media

For medium experiments, 11 commonly used laboratory media were selected with 1 × E3 as the control medium. The 11 solutions included 1 × E3, 1 × E3 with 0.05% methylene blue (1 × E3 MB), 1 × E2, 1 × E2 with 0.05% methylene blue (1 × E2 MB), 0.5 × E2 with 0.05% methylene blue (0.5 × E2 MB), Normal Ringer's solution (Ringer's), High calcium Ringer's (Hi Ca^2+^ Ringer's), Bath solution, 10% Hank's, Egg water, and system water from our laboratory's fish facility. Details about each medium, including solute concentrations, are reported in Supplementary Table S1.

### Behavioral assays and analyses

Larvae were tested at 5–6 dpf within their normal light cycle. Larvae were screened for morphological defects, including uninflated swim bladder, pericardial edema, and bent tail. For medium and *chd7^ncu101/^* experiments, swim bladder inflation and less severe morphological phenotypes (those not affecting mobility) were ignored.

Postscreening, larvae adapted to the testing lighting and temperature conditions for 30 min before placement in a custom laser-cut acrylic grid consisting of 36 circular wells, each with 9 mm diameter and 2 mm depth. For arena size experiments, 4 well diameters were tested: 9 mm (36 wells), 13 mm (16 wells), 18 mm (9 wells), and 28 mm (4 wells). All wells had 2 mm depth. The grid was anchored to a 60 × 5 × 5 mm aluminum rod attached to an acoustic shaker (Bruel and Kjaer). Spontaneous movement, acoustic startle, short-term habituation, and PPI assays were tracked and analyzed using FLOTE software, as previously described.^11,12,20^

### Spontaneous movement and thigmotaxis

Once individually placed in 9-mm round wells for all medium experiments, or 9-, 13-, 18-, and 28-mm wells for testing arena experiments, larvae acclimated for 3 min and were then recorded for 18.5 min at 50 fps at 640 × 640 px resolution with a Photron mini-UX50 high-speed camera. Thigmotaxis was analyzed by dividing the area of each well into its center and periphery, with each accounting for 50% of the total area and calculating the distance from center throughout the time course $\left(\surd \left[{\left(x-50\right)}^{2}+{\left(y-50\right)}^{2}\right]\right)$, where *x* and *y* represent coordinates of the larvae relative to the center of the arena. If the calculated distance from center was less than 25, or half of the center area, larvae were classified as in the center. If the distance from center was greater than 25, larvae were classified as in the perimeter.

### Acoustic startle response and auditory behaviors

Larvae acclimated for 2 min before auditory stimulus onset. Larvae received a total of 60 acoustic stimuli: 10 pseudorandomized trials of 6 intensities (13.6, 25.7, 29.2, 35.5, 39.6, and 53.6 dB), with a 20 s interstimulus interval (ISI). Immediately following the acoustic startle response assay, larvae received 10 trials of a prepulse stimulus at 29.2 dB and a pulse of 53.6 dB 300 ms later, with each trial separated by a 20 s ISI to assess PPI. At the end of the assay, larvae received 30 additional acoustic stimuli (53.6 dB) with a 1 s ISI to induce short-term habituation. Recordings were captured at 1000 fps and 640 × 640 px resolution with a Photron mini-UX50 high-speed camera.

### Lateral line/neuromast staining and imaging

Lateral line neuromasts were assessed using DASPEI (2-[4-(Dimethylamino)styryl]-N-ethylpyridinium iodide) (Fisher Scientific) stain. Wild-type TLF larvae were reared in selected media until 5 dpf. Larvae were submerged in 1 × E3 medium containing 0.007% DASPEI for 15 min at room temperature. Larvae were quickly washed with fresh 1 × E3 medium three times and anesthetized with 0.2% Tricaine. Larvae were laterally mounted in 1.5% low-melting point agarose in 1 × E3 medium and observed with a fluorescent dissecting microscope at 10 × total magnification (Nikon SMZ25). The total number of neuromasts was counted manually.

### Morphological phenotype assessment of *chd7^ncu101/^*

Larvae were assessed for morphological phenotypes after behavior testing. Larvae were anesthetized in 0.2% Tricaine (MS-222) and manually assessed by two independent experimenters, blinded to genotype. Morphological phenotypes were determined as previously described.^19^

### DNA extraction and genotyping *chd7^ncu101/^*

Larvae were individually placed in 96-well plates containing methanol (MeOH) for tissue fixation. DNA was extracted from whole larvae using a lysis buffer of 25 mM sodium hydroxide, 0.2 mM EDTA (base solution), and 40 mM Tris-HCl (neutralization solution). PCR and gel electrophoresis were performed as previously described.^19^

### Statistics

Statistical analyses were completed using Prism 8 (GraphPad) and JMP Pro 14 and JMP Pro 16 (SAS). All datasets were tested for normality using the Shapiro–Wilk test. One-way ANOVA compared to controls and multiple comparisons or nonparametric tests (Wilcoxon each pair) were used, respectively. For medium experiments, *p*-value threshold was set α = 0.01, and testing arena and *chd7^ncu101/^* experiments was α = 0.05. All data are represented as mean ± standard deviation, unless otherwise noted in the figure legends.

## Results

### Larger testing arenas cause acoustic hypo-responsiveness in zebrafish larvae

Some neuropsychiatric and congenital disorders such as schizophrenia and CHARGE syndrome commonly present with auditory-driven behavioral symptoms.^21,22^ Symptoms are typically in the form of dysregulated sensorimotor gating, and conductive/sensorineural hearing loss, respectively.^21–23^ Larval zebrafish are a robust model to study clinically relevant auditory-derived symptoms because by 5 dpf, larvae can perform multiple auditory-driven behaviors, including two types of acoustic startle response (short- and long-latency C-bends: SLC and LLCs), and multiple forms of startle modulation, including PPI, and short-term habituation.^11,24,25^

**FIG. 1.**
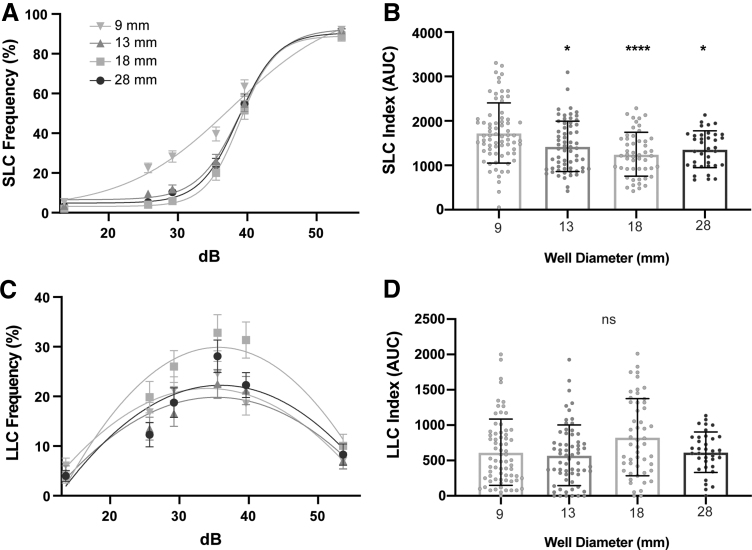
Testing arena size affects SLCs. **(A)** Acoustic startle responses, average SLC frequency comparing testing arena sizes (9 mm: *n* = 72; 13 mm: *n* = 64; 18 mm: *n* = 51; 28 mm: *n* = 36) (mean ± SEM). **(B)** SLC sensitivity index, calculated by the area under the SLC frequency curve for individual larvae (mean ± SD). **(C)** Average LLC frequency comparing testing arena sizes (mean ± SEM). **(D)** LLC sensitivity index, calculated by the area under the LLC frequency curve. *Asterisks* represent statistical significance for arena sizes compared to 9 mm arena (mean ± SD, Wilcoxon/Kruskal–Wallis tests with Wilcoxon each pair for nonparametric multiple comparisons, *p < 0.05, ****p < 0.0001). SLC, short-latency c-bend; LLC, long-latency c-bend; ns, not significantly different.

To determine the effect that testing arena diameter has on the acoustic startle response and its kinematics, we assayed 6 dpf wild-type TLF-strain larvae that were reared together in 1 × E3 medium in 10 cm Petri dishes and then tested individually in one of four testing arena sizes: 9, 13, 18, and 28 mm diameter circular wells. All arenas had a 2 mm depth. Larvae received 60 acoustic stimuli: 10 trials at each of 6 intensities, with a 20 s ISI. Following an acoustic stimulus, zebrafish larvae perform one of two distinct response types: (1) SLCs and (2) LLCs.^11^ SLCs and LLCs are defined by their response latency and kinematics and are mediated by separate neural circuits.^11,26^ As decibel value increases, larvae bias their responses toward SLCs, whereas LLCs are more likely to be performed following lower intensity stimuli^11^ (Fig. 1A).

To assess individual response rates of SLCs and LLCs, we calculated sensitivity indices by taking the area under the curve (AUC) for individual frequency curves (Fig. 1B, C). Compared to the 9 mm arena, larvae tested in all three larger arenas (13, 18, and 28 mm) showed a reduction in SLC frequency (Fig. 1A, B), but LLCs were unaffected (Fig. 1C, D). Statistical analyses for multiple comparisons are reported in Supplementary Table S2. The SLC response is a stereotyped series of movements following an intense acoustic stimulus, including an initial C-bend (C1), counter-bend (C2), and swim bout.^10–12^ Because of the highly stereotyped nature of the SLC response movements, we compared specific kinematic parameters of SLCs performed by larvae in different arena sizes, including latency of response initiation, bend angles (C1 and C2), C1 bend curvature, distance traveled, and C1 angular velocity (Supplementary Fig. S1).

The initial C-bend (C1) can be quantified using the angle (the change in head orientation), and curvature (the sum of angular changes in head orientation and tail position).^12^ Although we detected differences in SLC responsiveness, SLC kinematics were mostly unchanged, including latency, C1 curvature, and SLC distance (Supplementary Fig. S1A, C, D). We observed significant differences for C1 angle in the 13 mm arena (Supplementary Fig. S1B), and maximum angular velocity in the 13 and 18 mm arenas (Supplementary Fig. S1E). Larvae in the three largest arena sizes displayed significant increases in C2-bend angle (Supplementary Fig. S1F). This may be, at least partly, due to the fact that in a larger testing arena, larvae are less likely to contact the wall of the arena following the initial C1-bend.

### Activity levels and thigmotaxis behavior are dependent on arena size

**FIG. 2.**
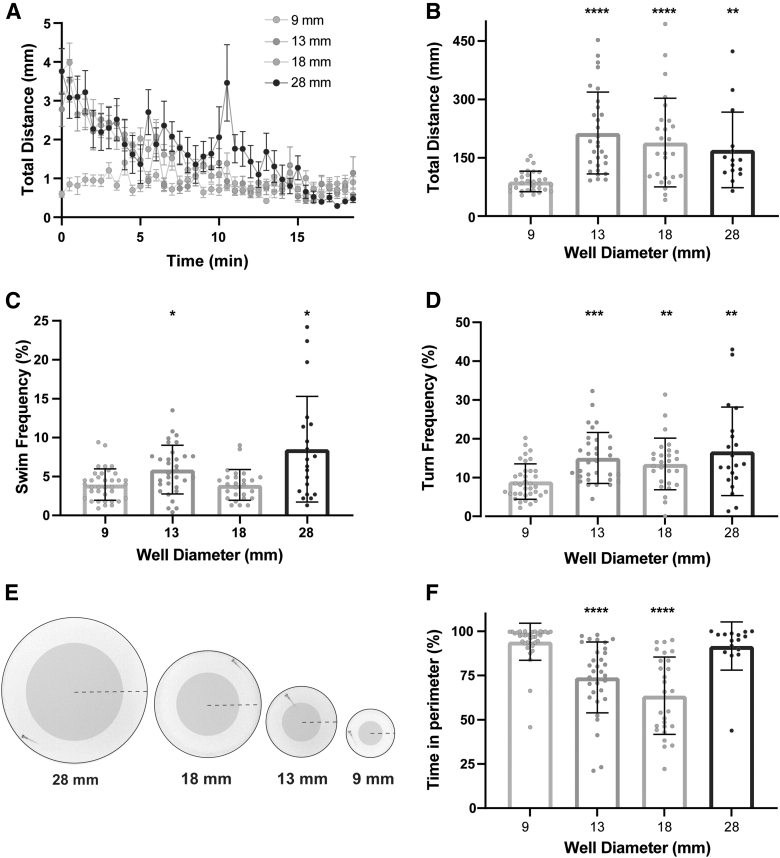
Arena size influences general locomotor activity and thigmotaxis behavior. **(A)** Total distance time plot during 18.5 min of recording comparing testing arena sizes (9 mm: *n* = 30; 13 mm: *n* = 30; 18 mm: *n* = 26; 28 mm: *n* = 20) (mean ± SEM). **(B)** Sum of distance traveled for individual larvae, and **(C)** swim and **(D)** turn frequencies (mean ± SD). **(E)** Images of each testing arena, with the *shaded area* indicating the center and the *non-shaded* area indicating the perimeter of the arena. The *dashed line* indicates the arena radius. **(F)** Percent of time spent in the perimeter during the 18.5 min testing period. *Asterisks* represent statistical significance for arena sizes compared to 9 mm arena (mean ± SD, Wilcoxon/Kruskal–Wallis tests with Wilcoxon Each Pair for nonparametric multiple comparisons, *p < 0.05, **p < 0.01, ***p < 0.001, ****p < 0.0001).

Previous work has demonstrated differences in overall activity levels of larval zebrafish as arena size changes,^17,27,28^ but to determine if arena size alters the types of movement performed and/or the location of larvae within the arena, we recorded and measured spontaneous locomotion at 50 frames per second for 18.5 min and analyzed total distance traveled, thigmotaxis behavior (preference for proximity to the wall), and frequency of swim and turn movements.^12,29^ Swim and turn frequencies are defined as the percentage of 5 s bins in which the larva performed a swim or turn. We found robust differences in total distance traveled as arena size increased (Fig. 2A, B), compared to the 9 mm arena. Larvae tested in 13 and 28 mm arenas displayed increased swim and turn frequencies (Fig. 2C, D), whereas the larvae in the 18 mm arena performed more turns, but similar swim frequency (Fig. 2D).

In addition to general locomotor behaviors, we analyzed thigmotaxis, defined as the percentage of time spent in the perimeter of the arena and a common measurement used in both rodent and zebrafish testing as an indicator of exploratory behavior and anxiety-like states.^30–32^ Center and perimeter were defined such that each accounted for 50% of the total area (Fig. 2E), and thus, if the larva had no preference, it would be equally likely to be in the center or perimeter.

Larvae in the 28 mm arena exhibited similar thigmotaxis rates compared to the 9 mm arena, while larvae in the 13 and 18 mm arenas spent significantly more time in the center (Fig. 2F). While larvae may be expected to spend the vast majority of their time in the perimeter of the smallest 9 mm arena since the well is only ∼2.5 times their body length, the increased thigmotaxis in the largest arena (28 mm) relative to the medium-sized 13- and 18-mm arenas suggests that larvae strongly prefer to remain close to the wall when exposed to a large, open space.

### Embryo medium influences auditory-driven behaviors and kinematics

Besides the testing arena size, embryo medium is another controllable variable that may influence larval behavior. To determine if medium type affects auditory-driven behavioral responses, we performed the same acoustic startle assay as above on larvae reared in 11 different types of commonly used media. We also grossly assessed morphological development and did not observe any overt difference in morphology of larvae raised in different media. To account for unequal sample sizes (Supplementary Table S3), we normalized startle sensitivity indices to 1 × E3, and data are presented as arbitrary units (AU).

**FIG. 3.**
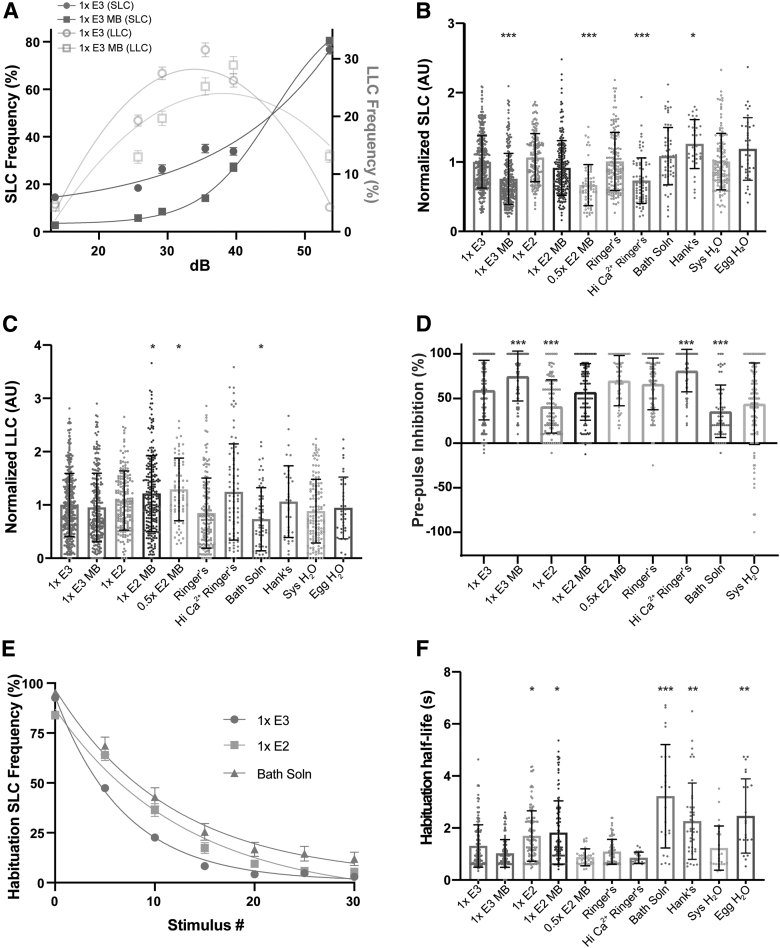
Embryo medium type impacts auditory-driven behaviors. **(A)** Acoustic startle responses, average SLC frequency (*left y*-axis), and LLC frequency (*right y*-axis) as acoustic stimulus intensity increases in 1 × E3 or 1 × E3 with methylene *blue*-treated larvae (mean ± SEM). **(B)** Normalized SLCs and **(C)** LLCs (AU), calculated by the area under the SLC or LLC frequency curves, respectively, for individual larvae, normalized to 1 × E3 (1 × E3: *n* = 397; 1 × E3 MB: *n* = 245; 1 × E2: *n* = 166; 1 × E2 MB: *n* = 231; 0.5 × E2 MB: *n* = 64; Ringer's: *n* = 141; Hi Ca^2+^ Ringer's: *n* = 79; Bath Solution: *n* = 61; Hank's: *n* = 36; Sys H_2_O: *n* = 123; Egg H_2_O: *n* = 36) (mean ± SD). **(D)** Rate of prepulse inhibition (1 × E3: *n* = 263; 1 × E3 MB: *n* = 118; 1 × E2: *n* = 108; 1 × E2 MB: *n* = 137; 0.5 × E2 MB: *n* = 63; Ringer's: *n* = 115; Hi Ca^2+^ Ringer's: *n* = 62; Bath Solution: *n* = 61; Sys H_2_O: *n* = 123) (mean ± SD). **(E)** Short-term habituation, average SLC frequency during 30 acoustic stimuli at highest intensity in 1 × E3-, 1 × E2-, and Bath Solution-treated larvae (mean ± SEM). **(F)** SLC half-life calculated by nonlinear regression (one-phase exponential decay) of SLC frequency curves for individual larvae. *Asterisks* represent statistical significance for medium type compared to 1 × E3 (mean ± SD, Wilcoxon/Kruskal–Wallis tests with Wilcoxon Each Pair for nonparametric multiple comparisons, *p < 0.01, **p < 0.001, ***p < 0.0001). AU, arbitrary unit.

Larvae reared in 1 × E3 MB, 0.5 × E2 MB, and Hi Ca^2+^ Ringer's displayed a significant reduction in SLC responses, while those raised in Hank's medium showed an increase in SLCs compared to 1 × E3 (Fig. 3A, B); 0.5 × E2 MB-reared larvae also performed more LLCs compared to 1 × E3 (Fig. 3C), indicating a shift in response type bias. In addition, 1 × E2 MB- and Bath solution-reared larvae displayed a significant increase or decrease in LLC responses, respectively (Fig. 3C). The independent effect of a medium on one of the two startle responses further illustrates the distinction between the two behaviors and suggests that the medium type may specifically impact the development and/or function of either the SLC or LLC circuit.

To further examine the influence media may have on sensorimotor gating, we performed a PPI assay. Larvae received 10 trials in which a weak stimulus (29.2 dB) was immediately followed by an intense stimulus (53.6 dB) 300 ms later. We calculated PPI% by comparing larval SLC response rates following an intense stimulus alone compared to the paired stimuli. Relative to larvae raised in 1 × E3, PPI was significantly increased in 1 × E3 MB and Hi Ca^2+^ Ringer's larvae, while 1 × E2 and Bath solution reduced PPI (Fig. 3D). Hank's and Egg H_2_O were not tested for PPI. Given that 1 × E3 MB and Hi Ca^2+^ Ringer's reduced SLC responses, an increase in PPI suggests these conditions suppress activity in the SLC circuit in both contexts.

Finally, we analyzed short-term habituation, a simple form of nonassociative learning in which SLC responses rapidly decrease following repeated strong acoustic stimuli.^24,25^ Larvae received 30 strong acoustic stimuli (53.6 dB) separated by a 1 s ISI (Fig. 3E). To measure the rate of habituation, we calculated the half-life of SLC frequency during the assay and found that larvae reared in 1 × E2, 1 × E2 MB, Bath solution, Hank's, and Egg H_2_O displayed slower habituation rates compared to 1 × E3. These assay- and context-dependent differences in acoustically driven behaviors suggest that embryo media can influence the development and/or function of neural circuits that control specific characteristics of these behavioral responses, rather than having a global effect on response rates.

Larvae raised in 1 × E3 MB, 0.5 × E2 MB, and Hi Ca^2+^ Ringer's initiated SLCs with significantly longer latencies, while Bath solution-, Hank's-, and Egg water-raised larvae responded with shorter latencies compared to 1 × E3 (Supplementary Fig. S2A). We found that larvae reared in Ringer's, Hi Ca^2+^ Ringer's, and Bath solution displayed a reduction in both C1 angle and C1 curvature (Supplementary Fig. S2B, C).

In addition, System H_2_O decreased C1 curvature, but not C1 head angle, compared to 1 × E3 (Supplementary Fig. S2B, C), indicating a possible specific effect on tail bending. 1 × E3 MB-, Ringer's- and Hi Ca^2+^ Ringer's-raised larvae traveled less distance during SLC responses compared to 1 × E3 (Supplementary Fig. S2D). 1 × E3 MB, 1 × E2 MB, 0.5 × E2 MB, Ringer's, Hi Ca^2+^ Ringer's, and Bath solution larvae all displayed reduced maximum angular velocities (Supplementary Fig. S2E), further illustrating specific kinematic differences resulting from the choice of media. Finally, larvae reared in 1 × E3 MB performed weaker counter-bends (C2), while Bath solution and System H_2_O increased C2 angle (Supplementary Fig. S2F). The medium-specific variation in SLC kinematic parameters suggests that the embryo medium can selectively influence the development and/or function of motor pathways that determine the performance of the response.

### Embryo medium alters spontaneous locomotor activity and behaviors

**FIG. 4.**
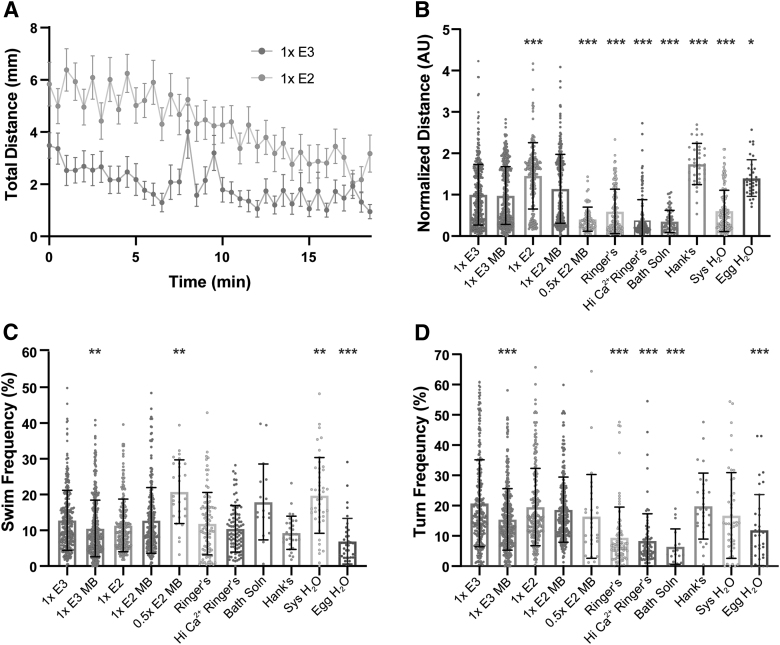
Medium type induces hypoactivity and hyperactivity in general locomotor behaviors. **(A)** Total distance time plot during 18.5 min of recording in 1 × E3- and 1 × E2-treated larvae (mean ± SEM). **(B)** Sum of distance traveled for individual larvae, normalized to 1 × E3 (1 × E3: *n* = 398; 1 × E3 MB: *n* = 382; 1 × E2: *n* = 193; 1 × E2 MB: *n* = 265; 0.5 × E2 MB: *n* = 72; Ringer's: *n* = 154; Hi Ca^2+^ Ringer's: *n* = 140; Bath Solution: *n* = 69; Hank's: *n* = 36; Sys H_2_O: *n* = 141; Egg H_2_O: *n* = 36). **(C)** Swim and **(D)** turn frequencies. *Asterisks* represent statistical significance for medium type compared to 1 × E3 (mean ± SD, Wilcoxon/Kruskal–Wallis tests with Wilcoxon Each Pair for nonparametric multiple comparisons, *p < 0.01, **p < 0.001, ***p < 0.0001).

To further investigate medium-specific influences on general locomotion, we recorded spontaneous locomotion and analyzed the frequency of swim and turn movements^12,29^ and total distance traveled (Fig. 4A). Normalized to 1 × E3 larvae, larvae raised in 1 × E2, Hank's, and Egg H_2_O were hyperactive and displayed greater distance traveled, while 0.5 × E2 MB, Ringer's, Hi Ca^2+^ Ringer's, Bath solution, and System H_2_O larvae were hypoactive during the 18.5-min recording period (Fig. 4B). Larvae reared in 1 × E3 MB displayed significantly reduced swim and turn frequencies (Fig. 4C, D), and while larvae in 1 × E2 moved significantly farther, their swim and turn frequencies were similar to 1 × E3 (Fig. 4C, D), suggesting more vigorous swims/turns that propelled them farther.

We also observed significantly increased swim frequencies in 0.5 × E2 MB and System H_2_O larvae, while Egg H_2_O larvae displayed a reduction (Fig. 4C). Like larvae reared in 1 × E2, Egg H_2_O and Hank's larvae, which were hyperactive, but showed a reduced frequency of swim bouts (Fig. 4C), likely traveled more distance per swim bout. Ringer's, Hi Ca^2+^ Ringer's, and Bath solution larvae performed fewer turns, largely consistent with their trend of decreased total movement (Fig. 4D). Taken together, our data show that embryo medium can impact nearly every aspect of auditory-driven and locomotor behaviors that we analyzed, including startle response threshold, bias, and modulation, as well as kinematic performance and spontaneous movement initiation.

### Medium type influences morphological and behavioral phenotypes in a CHARGE syndrome model

CHARGE syndrome is a rare and heterogeneous disorder characterized by a spectrum of physical and behavioral manifestations.^21,33,34^ The majority of CHARGE cases (∼70%) arises from *de novo* loss-of-function mutations in the chromatin remodeler, *Chromodomain-helicase-DNA-binding-protein-7* (CHD7).^35,36^ Previous work in our laboratory established a stable *chd7* mutant line (*chd7^ncu101/^*) in larval zebrafish to study the role of *chd7* in CHARGE-related sensorimotor behaviors.^19^ Homozygous *chd7* mutants recapitulate CHARGE-related behavioral and morphological phenotypes such as auditory responsiveness and craniofacial, ear, and heart abnormalities, but phenotype penetrance is influenced by genetic background and/or mutation location.^17^

To determine if an experimental variable such as medium type can affect CHARGE-related phenotype penetrance, we reared and tested *chd7^ncu101/^* and wild-type siblings in 5 medium types (1 × E3, 1 × E3 MB, 1 × E2, 1 × E2 MB, and Hank's). By 6 dpf, *chd7* homozygous mutants displayed multiple morphological phenotypes with varying frequencies across medium types (Table 1), including craniofacial abnormalities (highest frequency: 40% 1 × E2 MB), enlarged pericardial area (54% in 1 × E2), uninflated swim bladder (75% in 1 × E2 MB), and abnormal otoliths, or hard crystal-like structures within the otic vesicle^37,38^ (23% in 1 × E3). Fifty-eight percent of homozygous mutants reared in 1 × E3 displayed at least one morphological phenotype, while 77–81% of homozygous mutants displayed phenotypes in the other medium types.

**Table 1.** Medium Type Influences Morphological Phenotype Frequencies in *chd7* Mutants

		Craniofacial defect	Otolith defect	Pericardial edema	Noninflated swim bladder	Larvae with any morphological phenotype
1x E3	*chd7^+/+^*	1/18, 5.6%	—	1/18, 5.6%	—	2/18, 11.1%
	*chd7^ncu101/+^*	2/26, 7.7%	1/26, 3.8%	—	1/26, 3.8%	4/26, 15.4%
	*chd7^ncu101/ncu101^*	4/17, 23.5%	4/17, 23.5%	3/17, 23.5%	8/17, 47.1%	10/17, 58.8%
1x E3 MB	*chd7^+/+^*	1/18, 5.6%	—	1/18, 5.6%	—	2/18, 11.1%
	*chd7^ncu101/+^*	—	—	6/38, 15.8%	2/38, 5.3%	7/38, 18.4%
	*chd7^ncu101/ncu101^*	3/14, 21.4%	2/14, 14.3%	1/14, 7.1%	8/14, 57.1%	11/14, 78.6%
1x E2	*chd7^+/+^*	—	—	—	—	0/21
	*chd7^ncu101/+^*	—	—	4/38, 10.5%	1/38, 2.6%%	4/38, 10.5%
	*chd7^ncu101/ncu101^*	3/11, 27.3%	—	6/11, 54.5%	8/11, 72.7%	9/11, 81.8%
1x E2 MB	*chd7^+/+^*	—	—	—	—	0/13
	*chd7^ncu101/+^*	2/35, 5.7%	—	4/35, 11.4%	2/35, 5.7%	5/35, 14.3%
	*chd7^ncu101/ncu101^*	8/20, 40.0%	4/20, 20.0%	4/20, 20.0%	15/20, 75.0%	16/20, 80.0%
Hank's	*chd7^+/+^*	—	—	1/18, 5.6%	—	1/18, 5.6%
	*chd7^ncu101/+^*	—	—	2/33, 6.1%	1/33, 3.0%	2/33, 6.1%
	*chd7^ncu101/ncu101^*	6/18, 33.3%	1/18, 5.6%	4/18, 22.2%	10/18, 55.6%	14/18, 77.8%

Morphological phenotype ratio and frequencies in 6 dpf *chd7^+/+^, chd7^ncu101/+,^ and chd7^ncu101/ncu101^.* Explanation and discussion of phenotype presence in wild-type siblings have been previously described.^19^

To determine the impact of medium type on previously described behavioral phenotypes, we performed the acoustic startle response and spontaneous locomotor assays on *chd7^ncu101/^* and wild-type siblings. SLC and LLC sensitivity indices and distance traveled were normalized to *chd7^+/+^* in 1 × E3 and data are presented as AU. Following acoustic stimulation, *chd7^ncu101/ncu101^* show normal SLC response frequency, but reduced LLC response frequency,^19^ so we selected 5 media to test based on their influence on the acoustic startle response (Fig. 1).

**FIG. 5.**
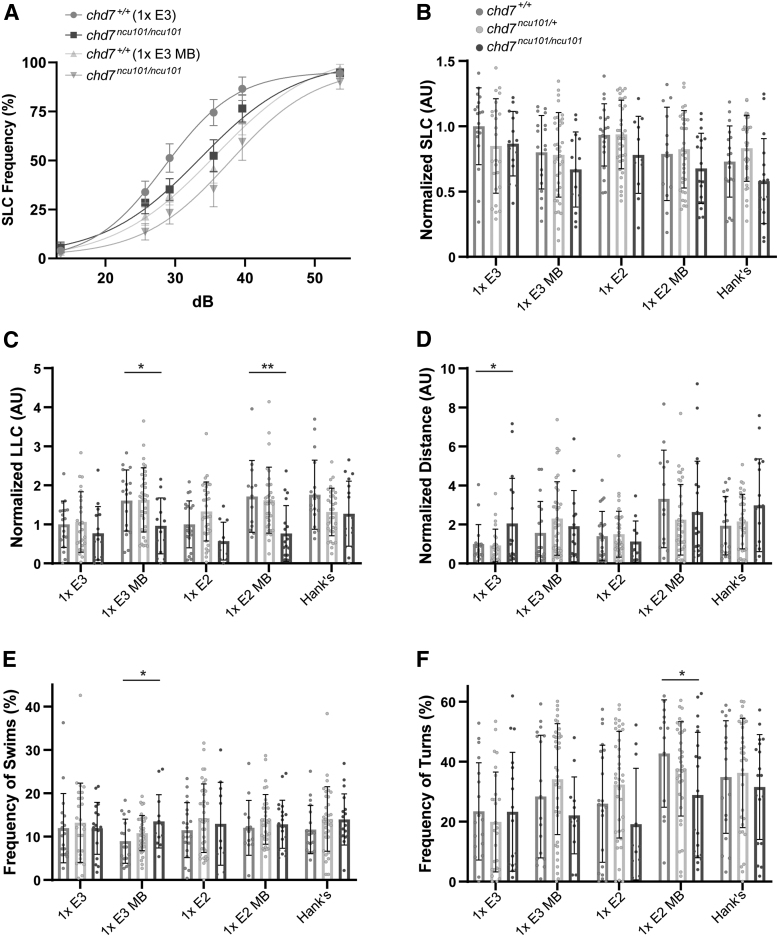
Medium type impacts behavioral phenotype penetrance in *chd7* mutants. **(A)** Acoustic startle responses, average SLC frequency of *chd7*^+/+^ and *chd7^ncu101/ncu101^* treated in 1 × E3 (*chd7^+/+^ n* = 18, *chd7^ncu101/ncu101^ n* = 15) or 1 × E3 with methylene *blue* (*chd7^+/+^ n* = 18, *chd7^ncu101/ncu101^ n* = 14) (mean ± SEM). **(B)** Normalized SLCs and **(C)** LLCs (AU), calculated by the area under the SLC or LLC frequency curves, respectively, for individual larvae, normalized to *chd7^+/+^* [1 × E3]. *Asterisks* represent statistical significance for *chd7^ncu101/+, ncu101/ncu101^* compared to wild-type siblings within medium type (1 × E3: *chd7^+/+^ n* = 18, *chd7^ncu101/+^ n* = 25, *chd7^ncu101/ncu101^ n* = 15; 1 × E3 MB: *chd7^+/+^ n* = 18*, chd7^ncu101/+^ n* = 38, *chd7^ncu101/ncu101^ n* = 14; 1 × E2: *chd7^+/+^ n* = 20*, chd7^ncu101/+^ n* = 36, *chd7^ncu101/ncu101^ n* = 11; 1 × E2 MB: *chd7^+/+^ n* = 13*, chd7^ncu101/+^ n* = 33, *chd7^ncu101/ncu101^ n* = 18; Hank's: *chd7^+/+^ n* = 18*, chd7^ncu101/+^ n* = 33, *chd7^ncu101/ncu101^ n* = 17). **(D)** Spontaneous locomotion, sum of total distance traveled, normalized to *chd7^+/+^* [1 × E3], **(E)** swim and **(F)** turn frequencies for individual larvae. *Asterisks* represent statistical significance for *chd7^ncu101/+, ncu101/ncu101^* compared to wild-type siblings within medium type (1 × E3: *chd7^+/+^ n* = 18*, chd7^ncu101/+^ n* = 26, *chd7^ncu101/ncu101^ n* = 17; 1 × E3 MB: *chd7^+/+^ n* = 18*, chd7^ncu101/+^ n* = 38, *chd7^ncu101/ncu101^ n* = 14; 1 × E2: *chd7^+/+^ n* = 21*, chd7^ncu101/+^ n* = 38, *chd7^ncu101/ncu101^ n* = 11; 1 × E2 MB: *chd7^+/+^ n* = 13*, chd7^ncu101/+^ n* = 26, *chd7^ncu101/ncu101^ n* = 20; Hank's: *chd7^+/+^ n* = 18*, chd7^ncu101/+^ n* = 33, *chd7^ncu101/ncu101^ n* = 18) (mean ± SD, One-way ANOVA with Student's *t* each pair test for multiple comparisons, *p < 0.05, **p < 0.01).

Wild-type siblings and homozygous mutants performed SLCs at similar rates, regardless of medium type (Fig. 5A, B), but mutants performed significantly fewer LLCs when reared in 1 × E3 MB and 1 × E2 MB, compared to wild-type siblings (Fig. 5C). Homozygous mutants in all other medium groups displayed a trend toward reduced LLCs, which did not reach statistical significance at the *p* < 0.05 level compared to wild-type siblings. We did find that when we compared wild types between medium groups (e.g., 1 × E3 wild type vs. 1 × E3 MB wild type), we observed the greatest number of differences compared to other genotypes (Supplementary Table S4).

This medium-dependent shift in responsiveness of wild types thus might mask the reduced LLC phenotype in *chd7* mutants. We saw similar results in spontaneous locomotor behaviors in which hyperactivity was seen in mutants reared in 1 × E3 compared to wild-type siblings (Fig. 5D), but there was a high degree of variability between the same genotypes (Supplementary Table S4). Mutants in 1 × E3 MB also performed more swims (Fig. 5E), while mutants in 1 × E2 MB performed fewer turns (Fig. 5F) compared to wild-type siblings, phenotypes previously not reported in the *chd7^ncu101/^* line. Taken together, these data show that medium type influences phenotype penetrance and may mask behavioral differences through genotype-specific effects in a zebrafish disease model.

## Discussion

In this study, we sought to define the impact of experimental variables such as testing arena size and embryo medium type on wild-type and mutant larval zebrafish behavior. As the use of larval zebrafish continues to expand in multiple fields in which behavior is often used as an experimental endpoint, including neuroscience, toxicology, and developmental biology, it is imperative to understand the influence experimental factors may have on results. In this study, we have demonstrated arena size- and medium-dependent differences in auditory and locomotor behaviors of larval zebrafish. In addition, medium type influences the expression of both morphological and behavioral phenotypes in a zebrafish CHARGE syndrome model in a genotype-specific manner. These results highlight the substantial impacts that these variables can have on experimental outcomes, and they underscore the importance of carefully designing studies to mitigate these impacts to ensure rigor and reproducibility in the zebrafish field.

A major advantage of the larval zebrafish model is that it is amenable to high-throughput testing, particularly in toxicological and neurobehavioral research.^6,14^ These studies typically require small-diameter wells and use locomotor behavior as an output for measuring toxicity. A few studies have reported significant changes in locomotion and activity levels when arena size is increased, while exposure concentrations and other variables remain constant.^17,27,28,39^

Another study reported significant changes in locomotion and an interaction between well size and the concentration of a neurostimulant.^28^ In these studies, locomotion of larvae was tested in dim light for an extended period,^39^ or in variations of the visuomotor response (VMR) assay in which locomotion is analyzed during repeated cycles of light and dark illumination.^17,27^ Although our study did not include this assay, our spontaneous locomotion data are consistent with these in showing that locomotion increases as arena size increases. In this study, we have expanded on this finding to demonstrate how arena size can also influence behavioral responses in multiple sensory modalities, highlighting the sensitivity of these behaviors to methodological variations.

In the acoustic startle response assay, larvae tested in the smallest arena size (9 mm diameter) were the most responsive, although this difference was exclusively for SLC and not LLC responses. It is not clear what may cause this hyperresponsiveness, but one possible explanation is that larvae may be more likely to be in contact with the wall of the smaller arena and therefore could receive both tactile and auditory stimulation. C2 bend angle was also decreased for larvae tested in the smallest arena. This is likely due to the confined space such that when larvae are close to the wall when the stimulus arrives, typically the C1 bend will be away from the wall and the C2 bend will turn larvae back toward the wall, sometimes hitting the wall and interrupting the full bend.

Larvae in larger arenas showed hyperactivity and increased turn frequencies, but thigmotaxis behavior was only decreased in medium-sized arenas (13 and 18 mm). Thigmotaxis or “wall-hugging” behavior has been previously characterized in 5 dpf zebrafish larvae and is thought to be a measure of exploratory behavior and anxiety-like states.^32^

Larvae in the 9 mm arenas exhibit strong thigmotaxis most likely because their overall activity is reduced and their space is limited, rather than resulting from an “anxiety-like” choice to stay at the perimeter and not explore the center. For the largest arena size (28 mm), although, overall activity was increased, while thigmotaxis was equivalent to that seen in the smallest arena. This heightened activity, while maintaining proximity to the wall, indicates a clear preference for avoiding the large open field. Taken together, these data indicate how arena size influences both spontaneous locomotor activity and stimulus-evoked responses. Overall, larger arenas may be more reliable for studies examining stimulus-evoked and spontaneous locomotor behaviors, as these exert fewer constraints on movement, although this would come at the expense of reduced throughput.

Another experimental factor we considered was the type of media in which larvae were raised. We selected 11 media that are commonly used in zebrafish laboratories. To account for observed differences purely due to sample size, we generated a comprehensive table of all the comparisons between media for each assay (Supplementary Table S3), and here we focus on what we believe to be most biologically relevant effects. Methylene blue is a synthetic cationic compound with a range of clinical and research applications, including as an antifungal agent and biocide in aquaculture.^40,41^ It is biologically active in zebrafish, and its toxicity in zebrafish cell cultures has been previously described.^41^ We observed significant differences in auditory and locomotor behaviors in larvae raised in 1 × E3 medium containing methylene blue (1 × E3 MB) compared to the 1 × E3 control.

Larvae raised in 1 × E3 MB were hyporesponsive in the acoustic startle response assay and showed increased PPI. Similar trends were observed when comparing 1 × E2 and 0.5 × E2 with methylene blue. These data show that methylene blue consistently suppresses the function of the SLC circuit. Methylene blue also caused significant changes in SLC kinematic performance (increased latency, reduced angular velocity), and these overall weakened responses also reflect suppressed activity in the startle circuit. A previous study found that 4 dpf larvae treated with varying concentrations of methylene blue were hypoactive during the VMR assay,^18^ which further illustrates the influence methylene blue has on stimulus-evoked behaviors and their underlying circuits.

A more recent study, however, did not report any effect of methylene blue exposure from 1 to 4 dpf on distance traveled in the VMR assay by 6 dpf larvae.^42^ This discrepancy could be because methylene blue was not present during testing and/or because it was used in 10% Hanks medium, a combination that we did not test. We did observe that methylene blue impacted some behaviors, but not others when in 1 × E3, and that it had a different set of impacts in 1 × E2. It is unlikely that the effects we observed were due to a higher dosage of methylene blue, since the concentration of methylene blue we used (1.5 μM) was at the low end of those tested by Hedge et al. (0.6–10 μM).^42^

On balance, it appears clear that methylene blue does affect some larval zebrafish behaviors, and that this may be dependent on the embryo medium used, timing of exposure, and the behavioral analysis methods. By using a high-speed camera, we were able to detect subtle differences in movement, which may not have been identified in other studies. Since methylene blue acts as an antifungal agent and biocide, it is possible that altered microbial makeup could contribute to the observed behavioral differences between fish raised with and without methylene blue. However, we did not observe any fungal growth in any of our medium conditions. Zebrafish larvae raised in germ-free conditions have been shown to display hyperactivity and modulation of stress-like behaviors,^43^ but the mechanisms driving behavioral differences in the presence or absence of causative microbes remain undetermined.

The presence of sodium bicarbonate (NaHCO_3_) and monopotassium phosphate (KH_2_PO_4_) defines the E2 medium, compared to E3. The impact of sodium bicarbonate and phosphate buffers has long been studied in the optimization of cell culturing techniques, including chick tissue and mouse embryos.^44,45^ The presence of NaHCO_3_ significantly increases the confluence of cells,^44^ while higher concentrations of KH_2_PO_4_ are detrimental to embryo development.^45,46^ Relatively low concentrations seem to provide the best environment for *in vitro* cell growth, but the effects of NaHCO_3_ past embryonic development *in vivo* are not well defined.

The E2 medium seemingly had no effect relative to 1 × E3 on the acoustic startle response and kinematics in zebrafish larvae, but did influence both PPI and short-term habituation. In addition, we observed hyperactivity in 1 × E2-raised larvae compared to 1 × E3. Although we cannot determine which component of E2 medium drives these behavioral differences, a contributing factor may be pH. The E2 medium has a slightly lower pH than E3 (pH 7.6 vs. pH 7.8), and changes in extracellular and intracellular H^+^ can impact neuronal function.^47^ Buffering agents sometimes added to embryo media such as HEPES, may stabilize pH, and thereby mitigate some of its effects, but our data do not provide a clear indication as to how and in what direction pH impacts larval behaviors. Alternatively, embryonic NaCOH_3_ exposure may have a developmental effect on behavior-specific circuits, rather than globally influencing sensorimotor behaviors.

Ringer's and High Calcium Ringer's contain higher Ca^2+^ concentrations compared to E2 and E3 media. Extracellular Ca^2+^ influences neuronal function and plays critical roles in synaptic transmission, neuronal survival, and axon growth.^48^ Since decreased extracellular Ca^2+^ generally increases neuronal excitability,^49^ it was not surprising to see multiple behavioral differences in higher Ca^2+^-containing solutions like High Calcium Ringer's. Larvae reared in High Calcium Ringer's performed fewer SLCs in the acoustic startle assay, and their responses were less kinematically robust. Higher Ca^2+^ concentrations caused an increase in PPI, but had no effect on short-term habituation, demonstrating the distinct regulatory mechanisms for these two types of behavioral modulation. Both Ringer's and High Calcium Ringer's caused a significant reduction in distance traveled and turn frequency compared to 1x E3, indicating they also impact the function of downstream motor pathways.

A possible explanation for the differences we observed is that some media may differentially affect sensory organ function, including hair cells of the ear and lateral line.^50^ However, we did not observe consistent change across all auditory responses with any one medium type, and so the selective differences we observed in distinct auditory-driven behaviors (SLCs and LLCs) and in the modulation of those behaviors during PPI and short-term habituation indicate that media more likely impact the development and/or function of specific behavioral circuits rather than that of hair cells.

Furthermore, we did not detect a difference in the number of lateral line neuromasts in wild-type larvae reared in a subset of media (Supplementary Fig. S3). In addition, the behavior most impacted by medium type was spontaneous locomotion, with 8 out of 11 altering total distance traveled relative to 1x E3. This robust change in response could reflect that distributed networks across the brain drive these behaviors, but it could also be a cautionary note to researchers about the sensitivity of this assay to different conditions. Together, our data indicate that media most likely produce their effects on larval zebrafish behavior by causing subtle changes in how the underlying neural circuits form and/or function, potentially through altered gene expression.

The medium and arena size experiments used wild-type TLF-strain larvae, but because larval zebrafish have become a powerful model for human genetic conditions, we also sought to define how embryo media might affect a mutant line with defined disease-related morphological and behavioral phenotypes. The *chd7* mutant line (*chd7^ncu101/^*) has previously been characterized to have variable phenotype penetrance,^19^ similar to the heterogeneous disorder it models, CHARGE syndrome.^51–53^ In this analysis, we found that medium type largely increased behavioral variance between wild types rather than heterozygotes and mutants, a finding that highlights how embryo media might mask some mutant phenotypes.

We also observed robust differences in specific morphological phenotypes in *chd7* mutants and in the overall penetrance of morphological phenotypes between medium groups. CHARGE syndrome is a very heterogeneous disorder, and external factors such as retinoic acid exposure and vitamin D deficiency can produce symptoms similar to those associated with CHARGE.^53–55^ In our study, methylene blue exposure may similarly contribute to morphological differences in *chd7* mutants. A previous study found impaired T cell development and possible immunological defects in *chd7* morphant and knockout models.^56^ The use of methylene blue or other biocide agents should thus be carefully considered when screening mutant lines with suspected immunological or developmental phenotypes.

Behavior can be highly variable between individuals, particularly in a nonisogenic model such as zebrafish. Both genetic and environmental factors contribute to behavioral variation, and so approaches like those used in this study could provide an opportunity to analyze gene by environment interactions. However, the lack of standardized methods in the field also makes it likely that a similar study using the same set of media would reveal different effects with another wild-type strain of zebrafish and/or in another laboratory setting with slightly different testing conditions. This underscores the importance of limiting experimental variation, of testing the robustness of observed phenotypes to such experimental variables, and – minimally – of rigorous documentation of experimental details to ensure that useful data and conclusions are reported, which advance our field.

## Conclusion

Analyzing the behavior of larval zebrafish is a versatile and powerful way to assess vertebrate neural development and function, but there is no standardized method for raising larvae and testing their behavior. In this study, we have demonstrated that two key experimental variables, embryo media and testing arena size, can exert significant effects on the performance of multiple conserved auditory and locomotor behaviors. We also show how different media can impact the expression of behavioral and developmental phenotypes in a zebrafish disease model. Our data add to a growing body of literature that highlights the importance of not only taking these experimental variables into account in designing larval zebrafish behavior studies but also of clearly reporting these methodological details to ensure rigor and reproducibility of results across laboratories.

## Supplementary Material

Supplemental data

Supplemental data

Supplemental data

Supplemental data

Supplemental data

Supplemental data

Supplemental data
