# Supplementary material for: Effects of 4 Testing Arena Sizes and 11 Types of Embryo Media on Sensorimotor Behaviors in Wild-Type and chd7 Mutant Zebrafish Larvae
Source: Zebrafish. 2024 Feb 14;21(1):1–14. doi: 10.1089/zeb.2023.0052 (PMC10902501; doi:10.1089/zeb.2023.0052)
Supplement: Supplemental data [file Suppl_TableS1.docx]

| **Media** | **Components** | **Abbreviation** |
| --- | --- | --- |
| 1x E3 | 5 mM NaCl, 0.17 mM KCl, 0.33 mM CaCl_2 *_ 2H_2_O,  0.33 mM MgSO_4_ | - |
| 1x E3, 0.05% methylene blue | 5 mM NaCl, 0.17 mM KCl, 0.33 mM CaCl_2 *_ 2H_2_O,  0.33 mM MgSO4, 0.5 mg/L methylene blue | 1x E3 MB |
| 1x E2 | 15 mM NaCl, 0.5 mM KCl, 1.0 mM MgSO_4_,  150 μM KH_2_PO_4_, 50 μM Na_2_HPO_4_, 1.0 mM CaCl_2,_ 0.7 mM NaHCO_3_ | - |
| 1x E2, 0.05% methylene blue | 15 mM NaCl, 0.5 mM KCl, 1.0 mM MgSO_4_, 150 μM KH_2_PO_4_,  50 μM Na_2_HPO_4_, 1.0 mM CaCl_2,_ 0.7 mM NaHCO_3_,  0.5 mg/L methylene blue | 1x E2 MB |
| 0.5x E2, 0.05% methylene blue | 7.5 mM NaCl, 0.25 mM KCl, 0.5 mM MgSO_4_, 75μM KH_2_PO_4_,  25 μM Na_2_HPO_4_, 0.5 mM CaCl_2,_ 0.35 mM NaHCO_3_,  0.5 mg/L methylene blue | 0.5x E2 MB |
| Normal Ringer’s | 116 mM NaCl, 2.9 mM KCl, 1.8 mM CaCl_2_, 5.0 mM HEPES | Ringer’s |
| High calcium Ringer’s | 116 mM NaCl, 2.9 mM KCl, 10.0 mM CaCl_2_, 5.0 mM HEPES | Hi Ca^2+^ Ringer’s |
| Bath solution | 112 mM NaCl, 5 mM HEPES, 2 mM CaCl_2_, 3 mM Glucose,  2 mM KCl, 1 mM MgCl_2_ | Bath soln |
| 10% Hank’s | 0.137 M NaCl, 5.4 mM KCl, 0.25 mM Na_2_HPO_4_, 0.44 mM KH_2_PO_4_, 1.3 mM CaCl_2_, 1.0 mM MgSO_4,_ 4.2 mM NaHCO_3_ | Hank’s |
| Egg water | 60μg/mL “Instant Ocean” in dH_2_O | Egg H_2_O |
| System water | pH= 7.59, conductivity- 595 μS, nitrate- 5 ppm, and  nitrite- 0 ppm | Sys H_2_O |

**Supplemental Table 1.** **List of media types and solute concentrations.** For each media type, pH was measured and ranged between 7.5-8.3. For system water, specific water quality details are listed.
