# Supplementary material for: Effects of 4 Testing Arena Sizes and 11 Types of Embryo Media on Sensorimotor Behaviors in Wild-Type and chd7 Mutant Zebrafish Larvae
Source: Zebrafish. 2024 Feb 14;21(1):1–14. doi: 10.1089/zeb.2023.0052 (PMC10902501; doi:10.1089/zeb.2023.0052)
Supplement: Supplemental data [file Suppl_TableS2.docx]

| **SLC Index** | | | | | | |
| --- | --- | --- | --- | --- | --- | --- |
| **Comparison** | **(n)** | **p-Value** | **Mean Dif** | **Std Error Dif** | **Lower CL** | **Upper CL** |
| 9 mm – 13 mm | 72, 64 | **0.0023** | 302.4097 | 97.835 | 109.591 | 495.2284 |
| 9 mm– 18 mm | 72, 51 | **<.0001** | 479.1065 | 104.2275 | 273.689 | 684.5239 |
| 9 mm – 28 mm | 72, 36 | **0.0018** | 367.5389 | 116.2453 | 138.436 | 596.6416 |
| 13 mm – 18 mm | 64, 51 | 0.0998 | 176.6968 | 106.8944 | -33.977 | 387.3702 |
| 13 mm – 28 mm | 64, 36 | 0.5836 | 65.1292 | 118.6424 | -168.698 | 298.9562 |
| 18 mm – 28 mm | 51, 36 | 0.5836 | 65.1292 | 118.6424 | -168.698 | 298.9562 |
| **LLC Index** | | | | | | |
| **Comparison** | **(n)** | **p-Value** | **Mean Dif** | **Std Error Dif** | **Lower CL** | **Upper CL** |
| 9 mm – 13 mm | 70, 64 | 0.5816 | 43.213 | 78.29139 | -111.096 | 197.5219 |
| 9 mm– 18 mm | 70, 51 | **0.0112** | 213.2141 | 83.34109 | 48.952 | 377.4757 |
| 9 mm – 28 mm | 70, 36 | 0.9928 | 0.8441 | 92.84387 | -182.147 | 183.8353 |
| 13 mm – 18 mm | 64, 51 | **0.0029** | 256.4271 | 84.97173 | 88.951 | 423.9026 |
| 13 mm – 28 mm | 64, 36 | 0.6409 | 44.0571 | 94.31035 | -141.824 | 229.9387 |
| 18 mm – 28 mm | 51, 36 | **0.0323** | 212.3699 | 98.54257 | 18.147 | 406.593 |
| **Latency** | | | | | | |
| **Comparison** | **(n)** | **p-Value** | **Mean Dif** | **Std Error Dif** | **Lower CL** | **Upper CL** |
| 9 mm – 13 mm | 71, 64 | 0.9424 | 0.017606 | 0.24341 | -0.4621 | 0.497307 |
| 9 mm– 18 mm | 71, 54 | 0.1571 | 0.362024 | 0.254988 | -0.1405 | 0.864543 |
| 9 mm – 28 mm | 71, 36 | 0.8753 | 0.045383 | 0.288936 | -0.52404 | 0.614806 |
| 13 mm – 18 mm | 64, 54 | 0.1471 | 0.37963 | 0.260942 | -0.13462 | 0.893883 |
| 13 mm – 28 mm | 64, 36 | 0.9249 | 0.027778 | 0.294204 | -0.55203 | 0.607583 |
| 18 mm – 28 mm | 54, 36 | 0.1814 | 0.407407 | 0.303853 | -0.19141 | 1.006227 |
| **C1 Angle** | | | | | | |
| **Comparison** | **(n)** | **p-Value** | **Mean Dif** | **Std Error Dif** | **Lower CL** | **Upper CL** |
| 9 mm – 13 mm | 71, 64 | **0.0149** | 5.475132 | 2.232163 | 1.07608 | 9.87418 |
| 9 mm– 18 mm | 71, 54 | 0.4838 | 1.640063 | 2.338338 | -2.96823 | 6.24836 |
| 9 mm – 28 mm | 71, 36 | 0.2418 | 3.109937 | 2.649657 | -2.11189 | 8.33177 |
| 13 mm – 18 mm | 64, 54 | 0.1104 | 3.835069 | 2.392943 | -0.88084 | 8.55098 |
| 13 mm – 28 mm | 64, 36 | **0.0017** | 8.585069 | 2.697969 | 3.26803 | 13.90211 |
| 18 mm – 28 mm | 54, 36 | 0.0897 | 4.75 | 2.786451 | -0.74142 | 10.24142 |
| **C1 Curvature** | | | | | | |
| **Comparison** | **(n)** | **p-Value** | **Mean Dif** | **Std Error Dif** | **Lower CL** | **Upper CL** |
| 9 mm – 13 mm | 71, 64 | 0.4739 | 2.552377 | 3.557781 | -4.45914 | 9.5639 |
| 9 mm– 18 mm | 71, 54 | 0.513 | 2.441836 | 3.727009 | -4.90319 | 9.78686 |
| 9 mm – 28 mm | 71, 36 | 0.7265 | 1.478873 | 4.223213 | -6.84405 | 9.8018 |
| 13 mm – 18 mm | 64, 54 | 0.1917 | 4.994213 | 3.814044 | -2.52234 | 12.51076 |
| 13 mm – 28 mm | 64, 36 | 0.3495 | 4.03125 | 4.300216 | -4.44343 | 12.50593 |
| 18 mm – 28 mm | 54, 36 | 0.8285 | 0.962963 | 4.441244 | -7.78965 | 9.71557 |
| **SLC Distance** | | | | | | |
| **Comparison** | **(n)** | **p-Value** | **Mean Dif** | **Std Error Dif** | **Lower CL** | **Upper CL** |
| 9 mm – 13 mm | 70, 64 | 0.4049 | 0.100446 | 0.120375 | -0.13679 | 0.337682 |
| 9 mm– 18 mm | 70, 54 | **0.0352** | 0.267113 | 0.126063 | 0.018668 | 0.515559 |
| 9 mm – 28 mm | 70, 36 | 0.5776 | 0.079613 | 0.14275 | -0.20172 | 0.360945 |
| 13 mm – 18 mm | 64, 54 | 0.1964 | 0.166667 | 0.128611 | -0.0868 | 0.420133 |
| 13 mm – 28 mm | 64, 36 | 0.8859 | 0.020833 | 0.145004 | -0.26494 | 0.306609 |
| 18 mm – 28 mm | 54, 36 | 0.2119 | 0.1875 | 0.14976 | -0.10765 | 0.482648 |
| **C1 Max Angular Velocity** | | | | | | |
| **Comparison** | **(n)** | **p-Value** | **Mean Dif** | **Std Error Dif** | **Lower CL** | **Upper CL** |
| 9 mm – 13 mm | 71, 64 | **0.0013** | 4.695095 | 1.443905 | 1.84951 | 7.540679 |
| 9 mm– 18 mm | 71, 54 | **0.0016** | 4.830029 | 1.512585 | 1.84909 | 7.810966 |
| 9 mm – 28 mm | 71, 36 | **0.0055** | 4.808204 | 1.713967 | 1.43039 | 8.186014 |
| 13 mm – 18 mm | 64, 54 | 0.9306 | 0.134934 | 1.547908 | -2.91561 | 3.185483 |
| 13 mm – 28 mm | 64, 36 | 0.9484 | 0.113109 | 1.745218 | -3.32629 | 3.552508 |
| 18 mm – 28 mm | 54, 36 | 0.9903 | 0.021825 | 1.802453 | -3.53037 | 3.574021 |
| **C2 Angle** | | | | | | |
| **Comparison** | **(n)** | **p-Value** | **Mean Dif** | **Std Error Dif** | **Lower CL** | **Upper CL** |
| 9 mm – 13 mm | 71, 64 | **0.0002** | 8.94124 | 2.327049 | 4.3552 | 13.52729 |
| 9 mm– 18 mm | 71, 54 | **<.0001** | 14.4314 | 2.437737 | 9.6272 | 19.23559 |
| 9 mm – 28 mm | 71, 36 | **<.0001** | 17.36659 | 2.76229 | 11.9228 | 22.81039 |
| 13 mm – 18 mm | 64, 54 | **0.0288** | 5.49016 | 2.494664 | 0.5738 | 10.40654 |
| 13 mm – 28 mm | 64, 36 | **0.0031** | 8.42535 | 2.812656 | 2.8823 | 13.96841 |
| 18 mm – 28 mm | 54, 36 | 0.3134 | 2.93519 | 2.904899 | -2.7897 | 8.66003 |
| **Total Distance (spontaneous)** | | | | | | |
| **Comparison** | **(n)** | **p-Value** | **Mean Dif** | **Std Error Dif** | **Lower CL** | **Upper CL** |
| 9 mm – 13 mm | 30, 30 | **<.0001** | 124.68 | 23.39292 | 78.2516 | 171.1085 |
| 9 mm– 18 mm | 30, 26 | **<.0001** | 100.2328 | 24.27598 | 52.0517 | 148.4139 |
| 9 mm – 28 mm | 30, 15 | **0.0056** | 81.234 | 28.65036 | 24.371 | 138.097 |
| 13 mm – 18 mm | 30, 26 | 0.3164 | 24.4472 | 24.27598 | -23.7339 | 72.6283 |
| 13 mm – 28 mm | 30, 15 | 0.1327 | 43.446 | 28.65036 | -13.417 | 100.309 |
| 18 mm – 28 mm | 26, 15 | 0.5193 | 18.9988 | 29.3758 | -39.304 | 77.3016 |
| **Swim Frequency** | | | | | | |
| **Comparison** | **(n)** | **p-Value** | **Mean Dif** | **Std Error Dif** | **Lower CL** | **Upper CL** |
| 9 mm – 13 mm | 36, 32 | **0.0293** | 1.926042 | 0.872447 | 0.19706 | 3.655026 |
| 9 mm– 18 mm | 36, 26 | 0.9663 | 0.039103 | 0.924206 | -1.79246 | 1.870661 |
| 9 mm – 28 mm | 36, 20 | **<.0001** | 4.541667 | 1.001471 | 2.55699 | 6.526347 |
| 13 mm – 18 mm | 32, 26 | **0.0405** | 1.965144 | 0.948119 | 0.08619 | 3.844094 |
| 13 mm – 28 mm | 32, 20 | **0.012** | 2.615625 | 1.023581 | 0.58713 | 4.644123 |
| 18 mm – 28 mm | 26, 20 | **<.0001** | 4.580769 | 1.068041 | 2.46416 | 6.697376 |
| **Turn Frequency** | | | | | | |
| **Comparison** | **(n)** | **p-Value** | **Mean Dif** | **Std Error Dif** | **Lower CL** | **Upper CL** |
| 9 mm – 13 mm | 36, 32 | **0.0007** | 6.099306 | 1.740799 | 2.6498 | 9.54881 |
| 9 mm– 18 mm | 36, 27 | **0.0143** | 4.541667 | 1.824137 | 0.92702 | 8.15632 |
| 9 mm – 28 mm | 36, 20 | **0.0002** | 7.785556 | 1.998242 | 3.82591 | 11.74521 |
| 13 mm – 18 mm | 32, 27 | 0.4072 | 1.557639 | 1.872361 | -2.15257 | 5.26785 |
| 13 mm – 28 mm | 32, 20 | 0.4108 | 1.68625 | 2.042359 | -2.36082 | 5.73332 |
| 18 mm – 28 mm | 27, 20 | 0.1277 | 3.243889 | 2.113841 | -0.94483 | 7.43261 |
| **Thigmotaxis** | | | | | | |
| **Comparison** | **(n)** | **p-Value** | **Mean Dif** | **Std Error Dif** | **Lower CL** | **Upper CL** |
| 9 mm – 13 mm | 36, 32 | **<.0001** | 20.18532 | 4.163798 | 11.9311 | 28.43956 |
| 9 mm– 18 mm | 36, 27 | **<.0001** | 30.55153 | 4.363134 | 21.9021 | 39.20093 |
| 9 mm – 28 mm | 36, 16 | 0.6301 | 2.4872 | 5.149343 | -7.7208 | 12.69517 |
| 13 mm – 18 mm | 32, 27 | **0.0225** | 10.36621 | 4.478479 | 1.4881 | 19.24427 |
| 13 mm – 28 mm | 32, 16 | **0.001** | 17.69812 | 5.247435 | 7.2957 | 28.10055 |
| 18 mm – 28 mm | 27, 16 | **<.0001** | 28.06433 | 5.406967 | 17.3456 | 38.78301 |

**Supplemental Table 2.** **Multiple comparisons between testing arena sizes.** Multiple comparisons and their reported values for the acoustic startle response (SLC and LLC Index), SLC kinematics (latency, C1 angle, C1 curvature, SLC distance, C1 max angular velocity, and C2 angle), and general locomotor behaviors (total distance, swim and turn frequency) with statistically significant p-values in bold (α=0.05, ANOVA with student’s t each pair test for multiple comparisons).
