# Supplementary material for: Effects of 4 Testing Arena Sizes and 11 Types of Embryo Media on Sensorimotor Behaviors in Wild-Type and chd7 Mutant Zebrafish Larvae
Source: Zebrafish. 2024 Feb 14;21(1):1–14. doi: 10.1089/zeb.2023.0052 (PMC10902501; doi:10.1089/zeb.2023.0052)
Supplement: Supplemental data [file Suppl_FigS1.docx]

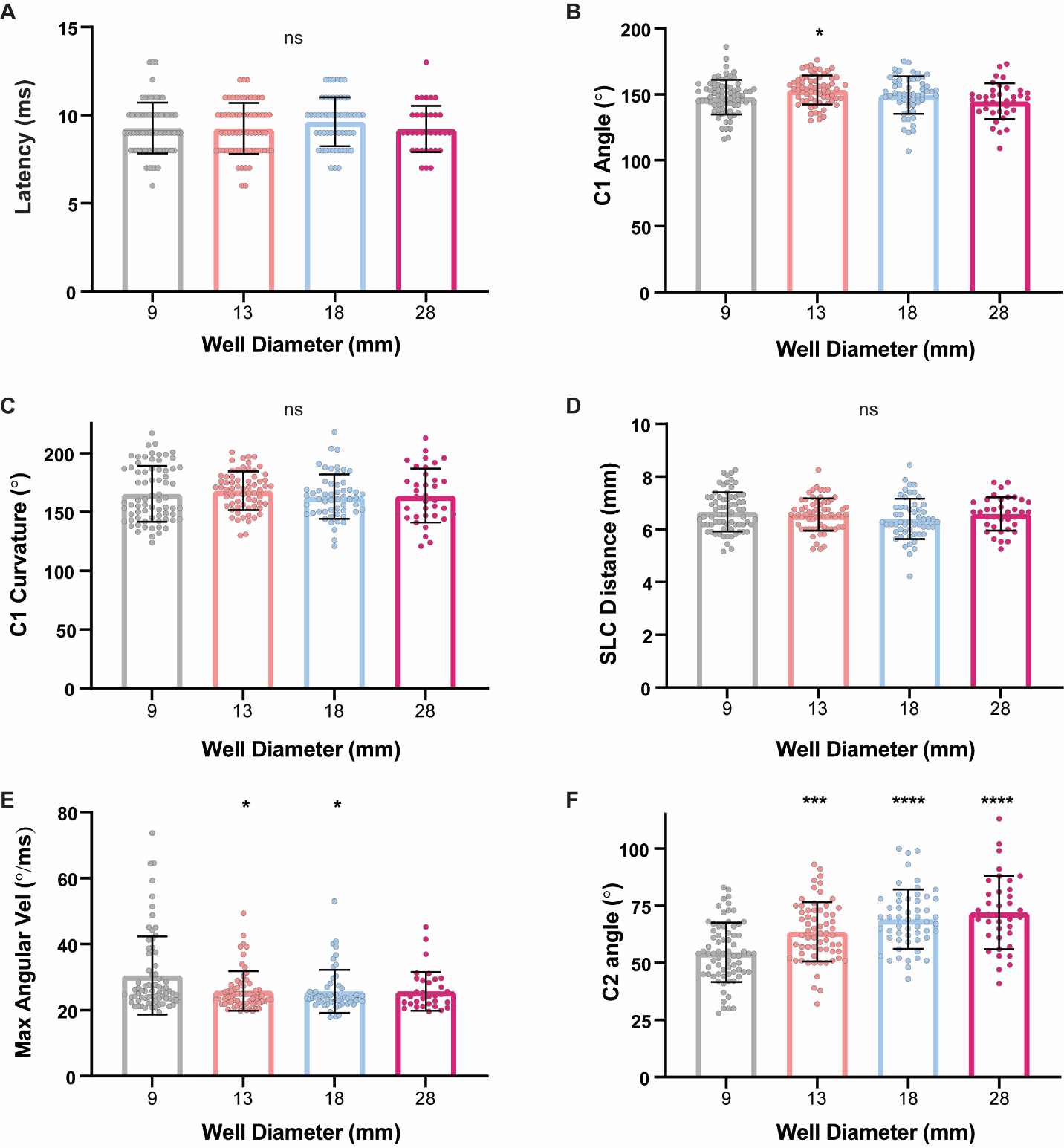


**Supplemental Figure 1.** **SLC kinematics compared across testing arena sizes. (A)** SLC response kinematics of individual larvae including latency of response initiation **(B)** C1 bend angle, **(C)** C1 bend curvature, **(D)** average distance traveled during response, **(E)** maximum angular velocity of C1 bend, and **(F)** C2 bend angle (9 mm: n=71; 13 mm: n=64; 18 mm: n=54; 28 mm: n=36). Asterisks represent statistical significance for arena sizes compared to 9 mm arena (mean ± SD, One-way ANOVA with student’s t each pair test for multiple comparisons, Wilcoxon/ Kruskal-Wallis tests with Wilcoxon Each Pair for nonparametric multiple comparisons, *p<0.05, ***p<0.001, ****p<0.0001).
