# Supplementary material for: Effects of 4 Testing Arena Sizes and 11 Types of Embryo Media on Sensorimotor Behaviors in Wild-Type and chd7 Mutant Zebrafish Larvae
Source: Zebrafish. 2024 Feb 14;21(1):1–14. doi: 10.1089/zeb.2023.0052 (PMC10902501; doi:10.1089/zeb.2023.0052)
Supplement: Supplemental data [file Suppl_FigS2.docx]

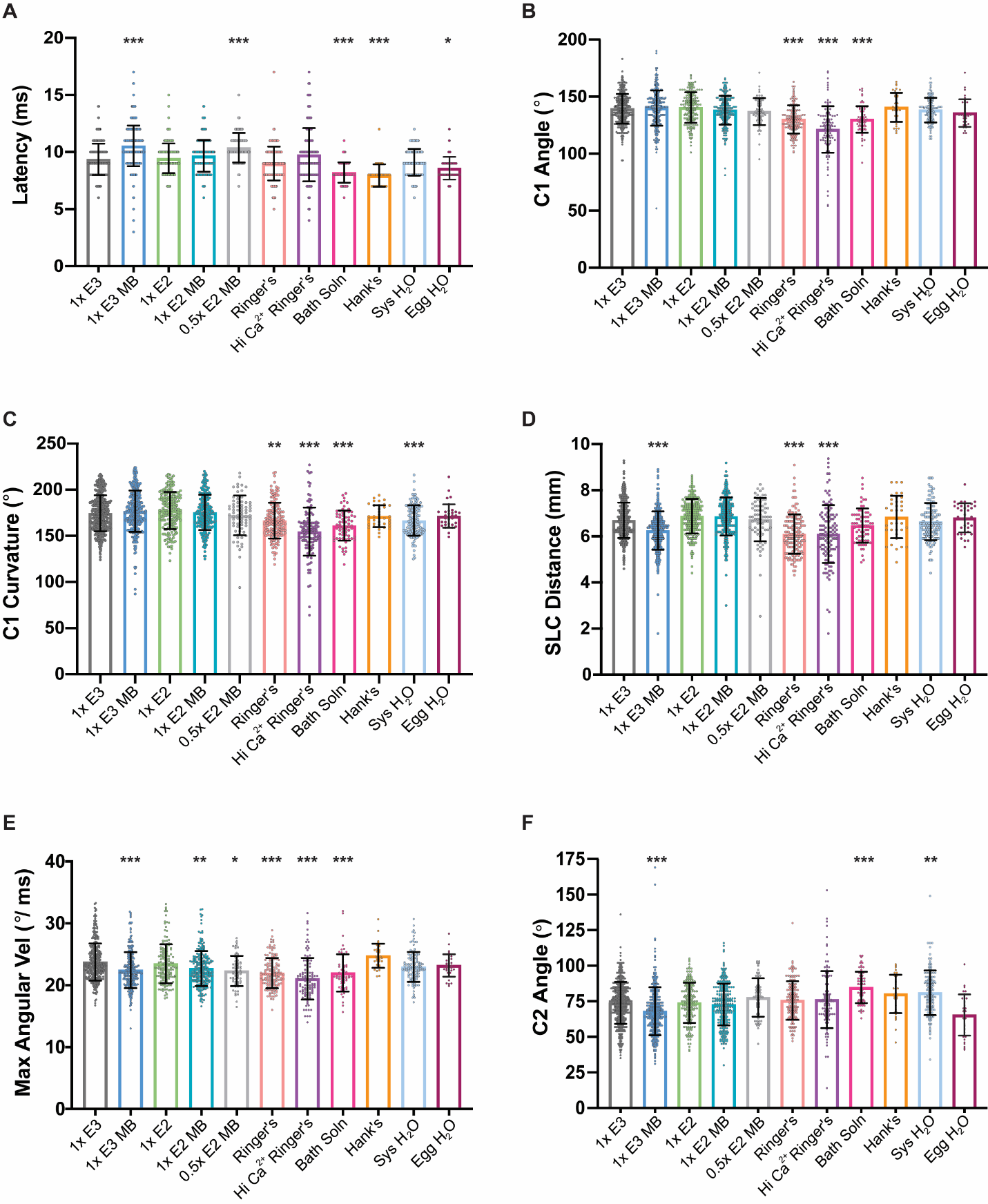


**Figure S2.** **SLC kinematics compared across media types. (A)** SLC response kinematics of individual larvae including latency of response initiation **(B)** C1 bend angle, **(C)** C1 bend curvature, **(D)** average distance traveled during response, **(E)** maximum angular velocity of C1 bend, and **(F)** C2 bend angle (1x E3: n= 380; 1x E3 MB: n=314; 1x E2: n=180; 1x E2 MB: n=250; 0.5x E2 MB: n=70; Ringer’s: n=150; Hi Ca^2+^ Ringer’s: n =120; Bath Soln: n=68; Hank’s: n=36; Sys H_2_O: n=138; Egg H_2_O: n=36). Asterisks represent statistical significance for media type compared to 1x E3 (mean ± SD, Wilcoxon/ Kruskal-Wallis tests with Wilcoxon Each Pair for nonparametric multiple comparisons, *p<0.01, **p<0.001, ***p<0.0001).
