# Supplementary material for: Effects of 4 Testing Arena Sizes and 11 Types of Embryo Media on Sensorimotor Behaviors in Wild-Type and chd7 Mutant Zebrafish Larvae
Source: Zebrafish. 2024 Feb 14;21(1):1–14. doi: 10.1089/zeb.2023.0052 (PMC10902501; doi:10.1089/zeb.2023.0052)
Supplement: Supplemental data [file Suppl_TableS4.docx]

| **Normalized SLC Index (by media)** | | | | | | |
| --- | --- | --- | --- | --- | --- | --- |
| **Comparison** | **(n)** | **p-Value** | **Mean Dif** | **Std Error Dif** | **Lower CL** | **Upper CL** |
| **1x E3** | | | | | | |
| *chd7^+/+^* – *chd7^ncu101/+^* | 18, 25 | 0.1281 | 0.150641 | 0.097499 | -0.04475 | 0.346034 |
| *chd7^+/+^* – *chd7^ncu101/ncu101^* | 18, 15 | 0.2328 | 0.133035 | 0.110268 | -0.08795 | 0.354017 |
| *chd7^ncu101/+^* – *chd7^ncu101/ncu101^* | 25, 15 | 0.8649 | 0.017606 | 0.103012 | -0.18884 | 0.224047 |
| **1x E3 MB** | | | | | | |
| *chd7^+/+^* – *chd7^ncu101/+^* | 18, 38 | 0.8215 | 0.019854 | 0.087656 | -0.15511 | 0.194817 |
| *chd7^+/+^* – *chd7^ncu101/ncu101^* | 18, 14 | 0.2309 | 0.131972 | 0.109167 | -0.08593 | 0.34987 |
| *chd7^ncu101/+^* – *chd7^ncu101/ncu101^* | 38, 14 | 0.2459 | 0.112118 | 0.095777 | -0.07905 | 0.30329 |
| **1x E2** | | | | | | |
| *chd7^+/+^* – *chd7^ncu101/+^* | 20, 36 | 0.9668 | 0.0030369 | 0.0727820 | -0.142362 | 0.1484356 |
| *chd7^+/+^* – *chd7^ncu101/ncu101^* | 20, 11 | 0.1228 | 0.1531875 | 0.0979638 | -0.042518 | 0.3488927 |
| *chd7^ncu101/+^* – *chd7^ncu101/ncu101^* | 36, 11 | 0.0871 | 0.1562244 | 0.0899078 | -0.023387 | 0.3358359 |
| **1x E2 MB** | | | | | | |
| *chd7^+/+^* – *chd7^ncu101/+^* | 13, 33 | 0.7226 | 0.035262 | 0.098862 | -0.16243 | 0.23295 |
| *chd7^+/+^* – *chd7^ncu101/ncu101^* | 13, 18 | 0.3169 | 0.110891 | 0.109889 | -0.10885 | 0.330627 |
| *chd7^ncu101/+^* – *chd7^ncu101/ncu101^* | 33, 18 | 0.1037 | 0.146154 | 0.088465 | -0.03074 | 0.323051 |
| **Hank’s** | | | | | | |
| *chd7^+/+^* – *chd7^ncu101/+^* | 18, 33 | 0.2155 | 0.101761 | 0.081352 | -0.06071 | 0.264232 |
| *chd7^+/+^* – *chd7^ncu101/ncu101^* | 18, 17 | 0.1153 | 0.149874 | 0.093897 | -0.03765 | 0.337399 |
| *chd7^ncu101/+^* – *chd7^ncu101/ncu101^* | 33, 17 | **0.0034** | 0.251635 | 0.082886 | 0.086101 | 0.41717 |
| **Normalized SLC Index (by genotype)** | | | | | | |
| **Comparison** | **(n)** | **p-Value** | **Mean Dif** | **Std Error Dif** | **Lower CL** | **Upper CL** |
| ***chd7^+/+^*** | | | | | | |
| 1x E3 – 1x E3 MB | 18, 18 | **0.0399** | 0.198976 | 0.095282 | 0.00943 | 0.388522 |
| 1x E3 – 1x E2 MB | 18, 13 | **0.0453** | 0.211491 | 0.104041 | 0.00452 | 0.418461 |
| 1x E3 – 1x E2 | 18, 20 | 0.4812 | 0.065719 | 0.092869 | -0.11903 | 0.250465 |
| 1x E3 – Hank’s | 18, 18 | **0.0057** | 0.270317 | 0.095282 | 0.080771 | 0.459862 |
| 1x E2 – 1x E3 MB | 20, 18 | 0.1551 | 0.133257 | 0.092869 | -0.05149 | 0.318003 |
| 1x E2 – 1x E2 MB | 20, 13 | 0.1561 | 0.145771 | 0.101836 | -0.05681 | 0.348356 |
| 1x E2 – Hank’s | 20, 18 | **0.0304** | 0.204598 | 0.092869 | 0.019851 | 0.389344 |
| 1x E3 MB – 1x E2 MB | 18, 13 | 0.9046 | 0.012515 | 0.104041 | -0.19446 | 0.219485 |
| 1x E3 MB – Hank’s | 18, 18 | 0.4562 | 0.071341 | 0.095282 | -0.11821 | 0.260886 |
| 1x E2 MB – Hank’s | 13, 18 | 0.5733 | 0.058826 | 0.104041 | -0.14814 | 0.265796 |
| ***chd7^ncu101/+^*** | | | | | | |
| 1x E3 – 1x E3 MB | 25, 38 | 0.3765 | 0.068189 | 0.07689 | -0.08366 | 0.22004 |
| 1x E3 – 1x E2 MB | 25, 33 | 0.747 | 0.025587 | 0.079168 | -0.13076 | 0.181936 |
| 1x E3 – 1x E2 | 25, 36 | 0.2595 | 0.087959 | 0.077733 | -0.06556 | 0.241474 |
| 1x E3 – Hank’s | 25, 33 | 0.8213 | 0.017914 | 0.079168 | -0.13843 | 0.174263 |
| 1x E2 – 1x E3 MB | 36, 38 | **0.0259** | 0.156148 | 0.069444 | 0.019003 | 0.293293 |
| 1x E2 – 1x E2 MB | 36, 33 | 0.1166 | 0.113546 | 0.071958 | -0.02856 | 0.255655 |
| 1x E2 – Hank’s | 36, 33 | 0.1432 | 0.105873 | 0.071958 | -0.03624 | 0.247983 |
| 1x E3 MB – 1x E2 MB | 38, 33 | 0.5496 | 0.042602 | 0.071046 | -0.09771 | 0.182912 |
| 1x E3 MB – Hank’s | 38, 33 | 0.4802 | 0.050275 | 0.071046 | -0.09004 | 0.190584 |
| 1x E2 MB – Hank’s | 33, 33 | 0.917 | 0.007673 | 0.073505 | -0.13749 | 0.152839 |
| ***chd7^ncu101/ncu101^*** | | | | | | |
| 1x E3 – 1x E3 MB | 15, 14 | **0.0668** | 0.197913 | 0.106273 | -0.01404 | 0.409867 |
| 1x E3 – 1x E2 MB | 15, 18 | **0.0624** | 0.189347 | 0.099979 | -0.01005 | 0.388747 |
| 1x E3 – 1x E2 | 15, 11 | 0.4519 | 0.085872 | 0.113521 | -0.14054 | 0.312282 |
| 1x E3 – Hank’s | 15, 17 | **0.006** | 0.287156 | 0.101306 | 0.085107 | 0.489205 |
| 1x E2 – 1x E3 MB | 11, 14 | 0.3342 | 0.112042 | 0.115224 | -0.11776 | 0.341848 |
| 1x E2 – 1x E2 MB | 11, 18 | 0.3477 | 0.103475 | 0.109446 | -0.11481 | 0.321757 |
| 1x E2 – Hank’s | 11, 17 | 0.0732 | 0.201284 | 0.11066 | -0.01942 | 0.421988 |
| 1x E3 MB – 1x E2 MB | 14, 18 | 0.9332 | 0.008567 | 0.101908 | -0.19468 | 0.211815 |
| 1x E3 MB – Hank’s | 14, 17 | 0.3902 | 0.089243 | 0.103211 | -0.1166 | 0.295089 |
| 1x E2 MB – Hank’s | 18, 17 | 0.3154 | 0.097809 | 0.096717 | -0.09509 | 0.290706 |
| **Normalized LLC Index (by media)** | | | | | | |
| **Comparison** | **(n)** | **p-Value** | **Mean Dif** | **Std Error Dif** | **Lower CL** | **Upper CL** |
| **1x E3** | | | | | | |
| *chd7^+/+^* – *chd7^ncu101/+^* | 18, 25 | 0.061601 | 0.216364 | -0.372 | 0.495205 | 0.7769 |
| *chd7^+/+^* – *chd7^ncu101/ncu101^* | 18, 15 | 0.229351 | 0.244699 | -0.26104 | 0.719738 | 0.3527 |
| *chd7^ncu101/+^* – *chd7^ncu101/ncu101^* | 25, 15 | 0.290952 | 0.228598 | -0.16717 | 0.749072 | 0.2085 |
| **1x E3 MB** | | | | | | |
| *chd7^+/+^* – *chd7^ncu101/+^* | 18, 38 | **0.016362** | 0.226689 | -0.43611 | 0.468836 | 0.9427 |
| *chd7^+/+^* – *chd7^ncu101/ncu101^* | 18, 14 | 0.649858 | 0.282319 | 0.086347 | 1.213369 | 0.0245 |
| *chd7^ncu101/+^* – *chd7^ncu101/ncu101^* | 38, 14 | 0.66622 | 0.247692 | 0.171826 | 1.160615 | 0.009 |
| **1x E2** | | | | | | |
| *chd7^+/+^* – *chd7^ncu101/+^* | 20, 36 | 0.0873 | 0.326871 | 0.188269 | -0.04924 | 0.70298 |
| *chd7^+/+^* – *chd7^ncu101/ncu101^* | 20, 11 | 0.0932 | 0.431854 | 0.253408 | -0.07439 | 0.938094 |
| *chd7^ncu101/+^* – *chd7^ncu101/ncu101^* | 36, 11 | **0.0018** | 0.758724 | 0.232569 | 0.294114 | 1.223334 |
| **1x E2 MB** | | | | | | |
| *chd7^+/+^* – *chd7^ncu101/+^* | 13, 33 | 0.09686 | 0.270983 | -0.445 | 0.638724 | 0.722 |
| *chd7^+/+^* – *chd7^ncu101/ncu101^* | 13, 18 | 0.949868 | 0.301207 | 0.347568 | 1.552168 | 0.0025 |
| *chd7^ncu101/+^* – *chd7^ncu101/ncu101^* | 33, 18 | 0.853008 | 0.242484 | 0.368131 | 1.337885 | 0.0008 |
| **Hank’s** | | | | | | |
| *chd7^+/+^* – *chd7^ncu101/+^* | 18, 33 | 0.442481 | 0.219014 | 0.005081 | 0.879882 | 0.0475 |
| *chd7^+/+^* – *chd7^ncu101/ncu101^* | 18, 17 | 0.487082 | 0.252786 | -0.01777 | 0.99193 | 0.0584 |
| *chd7^ncu101/+^* – *chd7^ncu101/ncu101^* | 33, 17 | **0.044601** | 0.223143 | -0.40105 | 0.490248 | 0.8422 |
| **Normalized LLC Index (by genotype)** | | | | | | |
| **Comparison** | **(n)** | **p-Value** | **Mean Dif** | **Std Error Dif** | **Lower CL** | **Upper CL** |
| ***chd7^+/+^*** | | | | | | |
| 1x E3 – 1x E3 MB | 18, 18 | **0.0175** | 0.610803 | 0.251841 | 0.109811 | 1.111796 |
| 1x E3 – 1x E2 MB | 18, 13 | **0.011** | 0.715283 | 0.274993 | 0.168235 | 1.262331 |
| 1x E3 – 1x E2 | 18, 20 | 0.9965 | 0.001075 | 0.245465 | -0.48723 | 0.489383 |
| 1x E3 – Hank’s | 18, 18 | **0.0034** | 0.75893 | 0.251841 | 0.257937 | 1.259923 |
| 1x E2 – 1x E3 MB | 20, 18 | **0.015** | 0.609728 | 0.245465 | 0.121421 | 1.098035 |
| 1x E2 – 1x E2 MB | 20, 13 | **0.0096** | 0.714208 | 0.269165 | 0.178753 | 1.249663 |
| 1x E2 – Hank’s | 20, 18 | **0.0028** | 0.757855 | 0.245465 | 0.269547 | 1.246162 |
| 1x E3 MB – 1x E2 MB | 18, 13 | 0.705 | 0.10448 | 0.274993 | -0.44257 | 0.651528 |
| 1x E3 MB – Hank’s | 18, 18 | 0.558 | 0.148127 | 0.251841 | -0.35287 | 0.649119 |
| 1x E2 MB – Hank’s | 13, 18 | 0.8743 | 0.043647 | 0.274993 | -0.5034 | 0.590695 |
| ***chd7^ncu101/+^*** | | | | | | |
| 1x E3 – 1x E3 MB | 25, 38 | **0.0047** | 0.565564 | 0.197473 | 0.175575 | 0.955554 |
| 1x E3 – 1x E2 MB | 25, 33 | **0.0069** | 0.556822 | 0.203323 | 0.155279 | 0.958364 |
| 1x E3 – 1x E2 | 25, 36 | 0.1841 | 0.266345 | 0.199638 | -0.12792 | 0.660609 |
| 1x E3 – Hank’s | 25, 33 | 0.2119 | 0.254847 | 0.203323 | -0.1467 | 0.65639 |
| 1x E2 – 1x E3 MB | 36, 38 | 0.0954 | 0.29922 | 0.178349 | -0.053 | 0.651442 |
| 1x E2 – 1x E2 MB | 36, 33 | 0.118 | 0.290477 | 0.184806 | -0.0745 | 0.65545 |
| 1x E2 – Hank’s | 36, 33 | 0.9505 | 0.011497 | 0.184806 | -0.35348 | 0.37647 |
| 1x E3 MB – 1x E2 MB | 38, 33 | 0.9618 | 0.008743 | 0.182465 | -0.35161 | 0.369093 |
| 1x E3 MB – Hank’s | 38, 33 | 0.0905 | 0.310717 | 0.182465 | -0.04963 | 0.671067 |
| 1x E2 MB – Hank’s | 33, 33 | 0.1117 | 0.301974 | 0.18878 | -0.07085 | 0.674797 |
| ***chd7^ncu101/ncu101^*** | | | | | | |
| 1x E3 – 1x E3 MB | 15, 14 | 0.4736 | 0.190296 | 0.264125 | -0.33649 | 0.717076 |
| 1x E3 – 1x E2 MB | 15, 18 | 0.9833 | 0.005234 | 0.248482 | -0.49035 | 0.500816 |
| 1x E3 – 1x E2 | 15, 11 | 0.4776 | 0.201428 | 0.28214 | -0.36128 | 0.764138 |
| 1x E3 – Hank’s | 15, 17 | 0.0504 | 0.501199 | 0.251782 | -0.00097 | 1.003363 |
| 1x E2 – 1x E3 MB | 11, 14 | 0.1757 | 0.391723 | 0.286372 | -0.17943 | 0.962873 |
| 1x E2 – 1x E2 MB | 11, 18 | 0.4731 | 0.196193 | 0.272011 | -0.34632 | 0.738702 |
| 1x E2 – Hank’s | 11, 17 | **0.0128** | 0.702626 | 0.275029 | 0.154098 | 1.251154 |
| 1x E3 MB – 1x E2 MB | 14, 18 | 0.4427 | 0.19553 | 0.253277 | -0.30961 | 0.700674 |
| 1x E3 MB – Hank’s | 14, 17 | 0.2296 | 0.310903 | 0.256515 | -0.2007 | 0.822506 |
| 1x E2 MB – Hank’s | 18, 17 | **0.0387** | 0.506433 | 0.240377 | 0.027016 | 0.98585 |
| **Normalized Total Distance (by media)** | | | | | | |
| **Comparison** | **(n)** | **p-Value** | **Mean Dif** | **Std Error Dif** | **Lower CL** | **Upper CL** |
| **1x E3** | | | | | | |
| *chd7^+/+^* – *chd7^ncu101/+^* | 18, 26 | 0.8604 | 0.077804 | 0.440424 | -0.8038 | 0.959409 |
| *chd7^+/+^* – *chd7^ncu101/ncu101^* | 18, 17 | **0.0341** | 1.054542 | 0.485782 | 0.082144 | 2.026939 |
| *chd7^ncu101/+^* – *chd7^ncu101/ncu101^* | 26, 17 | **0.0142** | 1.132346 | 0.448013 | 0.23555 | 2.029142 |
| **1x E3 MB** | | | | | | |
| *chd7^+/+^* – *chd7^ncu101/+^* | 18, 38 | 0.1562 | 0.741691 | 0.517177 | -0.2906 | 1.77398 |
| *chd7^+/+^* – *chd7^ncu101/ncu101^* | 18, 14 | 0.5891 | 0.349571 | 0.644092 | -0.93604 | 1.635183 |
| *chd7^ncu101/+^* – *chd7^ncu101/ncu101^* | 38, 14 | 0.4901 | 0.392121 | 0.565091 | -0.73581 | 1.520048 |
| **1x E2** | | | | | | |
| *chd7^+/+^* – *chd7^ncu101/+^* | 21, 38 | 0.7581 | 0.099976 | 0.323235 | -0.5452 | 0.745155 |
| *chd7^+/+^* – *chd7^ncu101/ncu101^* | 21, 11 | 0.5294 | 0.279752 | 0.442448 | -0.60338 | 1.162882 |
| *chd7^ncu101/+^* – *chd7^ncu101/ncu101^* | 38, 11 | 0.3542 | 0.379728 | 0.407008 | -0.43266 | 1.19212 |
| **1x E2 MB** | | | | | | |
| *chd7^+/+^* – *chd7^ncu101/+^* | 13, 36 | 0.138 | 1.06754 | 0.711026 | -0.35207 | 2.487149 |
| *chd7^+/+^* – *chd7^ncu101/ncu101^* | 13, 20 | 0.3914 | 0.675402 | 0.782854 | -0.88762 | 2.23842 |
| *chd7^ncu101/+^* – *chd7^ncu101/ncu101^* | 36, 20 | 0.5245 | 0.392137 | 0.612827 | -0.83141 | 1.615687 |
| **Hank’s** | | | | | | |
| *chd7^+/+^* – *chd7^ncu101/+^* | 18, 33 | 0.6695 | 0.216849 | 0.505781 | -0.79298 | 1.226674 |
| *chd7^+/+^* – *chd7^ncu101/ncu101^* | 18, 18 | 0.0729 | 1.048604 | 0.575373 | -0.10017 | 2.197373 |
| *chd7^ncu101/+^* – *chd7^ncu101/ncu101^* | 33, 18 | 0.1048 | 0.831755 | 0.505781 | -0.17807 | 1.841579 |
| **Normalized Total Distance (by genotype)** | | | | | | |
| **Comparison** | **(n)** | **p-Value** | **Mean Dif** | **Std Error Dif** | **Lower CL** | **Upper CL** |
| ***chd7^+/+^*** | | | | | | |
| 1x E3 – 1x E3 MB | 18, 18 | 0.2871 | 0.563367 | 0.525822 | -0.48247 | 1.609205 |
| 1x E3 – 1x E2 MB | 18, 13 | **0.0001** | 2.309603 | 0.57416 | 1.16762 | 3.451585 |
| 1x E3 – 1x E2 | 18, 21 | 0.4326 | 0.39956 | 0.506695 | -0.60824 | 1.407355 |
| 1x E3 – Hank’s | 18, 18 | 0.0802 | 0.931303 | 0.525822 | -0.11454 | 1.977142 |
| 1x E2 – 1x E3 MB | 21, 18 | 0.7473 | 0.163807 | 0.506695 | -0.84399 | 1.171602 |
| 1x E2 – 1x E2 MB | 21, 13 | **0.0009** | 1.910044 | 0.556696 | 0.8028 | 3.01729 |
| 1x E2 – Hank’s | 21, 18 | 0.297 | 0.531744 | 0.506695 | -0.47605 | 1.539539 |
| 1x E3 MB – 1x E2 MB | 18, 13 | **0.0032** | 1.746237 | 0.57416 | 0.60426 | 2.888218 |
| 1x E3 MB – Hank’s | 18, 18 | 0.486 | 0.367937 | 0.525822 | -0.6779 | 1.413776 |
| 1x E2 MB – Hank’s | 13, 18 | **0.0186** | 1.3783 | 0.57416 | 0.23632 | 2.520281 |
| ***chd7^ncu101/+^*** | | | | | | |
| 1x E3 – 1x E3 MB | 26, 38 | **0.0004** | 1.382862 | 0.383587 | 0.625523 | 2.1402 |
| 1x E3 – 1x E2 MB | 26, 36 | **0.0008** | 1.319868 | 0.387892 | 0.554031 | 2.085705 |
| 1x E3 – 1x E2 | 26, 38 | 0.1342 | 0.57734 | 0.383587 | -0.18 | 1.334679 |
| 1x E3 – Hank’s | 26, 33 | **0.0023** | 1.225957 | 0.395217 | 0.445658 | 2.006255 |
| 1x E2 – 1x E3 MB | 38, 38 | **0.021** | 0.805522 | 0.345761 | 0.122866 | 1.488177 |
| 1x E2 – 1x E2 MB | 38, 36 | **0.0356** | 0.742528 | 0.35053 | 0.050456 | 1.4346 |
| 1x E2 – Hank’s | 38, 33 | 0.0723 | 0.648616 | 0.358619 | -0.05943 | 1.356658 |
| 1x E3 MB – 1x E2 MB | 38, 36 | 0.8576 | 0.062994 | 0.35053 | -0.62908 | 0.755066 |
| 1x E3 MB – Hank’s | 38, 33 | 0.6623 | 0.156905 | 0.358619 | -0.55114 | 0.864947 |
| 1x E2 MB – Hank’s | 36, 33 | 0.7963 | 0.093911 | 0.363219 | -0.62321 | 0.811036 |
| ***chd7^ncu101/ncu101^*** | | | | | | |
| 1x E3 – 1x E3 MB | 17, 14 | 0.8593 | 0.141605 | 0.796137 | -1.44438 | 1.72759 |
| 1x E3 – 1x E2 MB | 17, 20 | 0.4282 | 0.579659 | 0.727707 | -0.87001 | 2.029326 |
| 1x E3 – 1x E2 | 17, 11 | 0.277 | 0.934734 | 0.853598 | -0.76572 | 2.635189 |
| 1x E3 – Hank’s | 17, 18 | 0.2187 | 0.925365 | 0.746051 | -0.56084 | 2.411575 |
| 1x E2 – 1x E3 MB | 11, 14 | 0.3751 | 0.793129 | 0.888802 | -0.97745 | 2.563713 |
| 1x E2 – 1x E2 MB | 11, 20 | **0.0714** | 1.514393 | 0.828066 | -0.1352 | 3.163984 |
| 1x E2 – Hank’s | 11, 18 | **0.0306** | 1.860099 | 0.844232 | 0.1783 | 3.541895 |
| 1x E3 MB – 1x E2 MB | 14, 20 | 0.3511 | 0.721264 | 0.768698 | -0.81006 | 2.252589 |
| 1x E3 MB – Hank’s | 14, 18 | 0.1788 | 1.06697 | 0.786086 | -0.49899 | 2.632933 |
| 1x E2 MB – Hank’s | 20, 18 | 0.631 | 0.345706 | 0.716697 | -1.08203 | 1.77344 |
| **Swim Frequency (by media)** | | | | | | |
| **Comparison** | **(n)** | **p-Value** | **Mean Dif** | **Std Error Dif** | **Lower CL** | **Upper CL** |
| **1x E3** | | | | | | |
| *chd7^+/+^* – *chd7^ncu101/+^* | 18, 26 | 0.6184 | 1.235043 | 2.466323 | -3.70184 | 6.171924 |
| *chd7^+/+^* – *chd7^ncu101/ncu101^* | 18, 17 | 0.989 | 0.037582 | 2.720319 | -5.40773 | 5.482892 |
| *chd7^ncu101/+^* – *chd7^ncu101/ncu101^* | 26, 17 | 0.6139 | 1.272624 | 2.508821 | -3.74933 | 6.294575 |
| **1x E3 MB** | | | | | | |
| *chd7^+/+^* – *chd7^ncu101/+^* | 18, 38 | 0.1812 | 1.847368 | 1.366933 | -0.8818 | 4.576538 |
| *chd7^+/+^* – *chd7^ncu101/ncu101^* | 18, 14 | **0.0105** | 4.580769 | 1.738818 | 1.10911 | 8.052432 |
| *chd7^ncu101/+^* – *chd7^ncu101/ncu101^* | 38, 14 | 0.0796 | 2.733401 | 1.53498 | -0.33129 | 5.798088 |
| **1x E2** | | | | | | |
| *chd7^+/+^* – *chd7^ncu101/+^* | 21, 38 | 0.1315 | 3.245739 | 2.125951 | -0.99768 | 7.489155 |
| *chd7^+/+^* – *chd7^ncu101/ncu101^* | 21, 11 | 0.5042 | 1.954113 | 2.910031 | -3.85433 | 7.762559 |
| *chd7^ncu101/+^* – *chd7^ncu101/ncu101^* | 38, 11 | 0.631 | 1.291627 | 2.676939 | -4.05157 | 6.63482 |
| **1x E2 MB** | | | | | | |
| *chd7^+/+^* – *chd7^ncu101/+^* | 13, 36 | 0.3049 | 1.954525 | 1.889697 | -1.82058 | 5.729629 |
| *chd7^+/+^* – *chd7^ncu101/ncu101^* | 13, 20 | 0.6911 | 0.824231 | 2.064547 | -3.30018 | 4.948638 |
| *chd7^ncu101/+^* – *chd7^ncu101/ncu101^* | 36, 20 | 0.4914 | 1.130294 | 1.63304 | -2.13208 | 4.392667 |
| **Hank’s** | | | | | | |
| *chd7^+/+^* – *chd7^ncu101/+^* | 18, 33 | 0.2111 | 2.44798 | 1.938642 | -1.42264 | 6.318604 |
| *chd7^+/+^* – *chd7^ncu101/ncu101^* | 18, 18 | 0.2916 | 2.344444 | 2.205386 | -2.05875 | 6.747639 |
| *chd7^ncu101/+^* – *chd7^ncu101/ncu101^* | 33, 18 | 0.9576 | 0.103535 | 1.938642 | -3.76709 | 3.974159 |
| **Swim Frequency (by genotype)** | | | | | | |
| **Comparison** | **(n)** | **p-Value** | **Mean Dif** | **Std Error Dif** | **Lower CL** | **Upper CL** |
| ***chd7^+/+^*** | | | | | | |
| 1x E3 – 1x E3 MB | 18, 18 | 0.1615 | 3.011111 | 2.13166 | -1.22868 | 7.250898 |
| 1x E3 – 1x E2 MB | 18, 13 | 0.9762 | 0.069658 | 2.32762 | -4.55989 | 4.699202 |
| 1x E3 – 1x E2 | 18, 21 | 0.6444 | 0.951587 | 2.054119 | -3.13397 | 5.037148 |
| 1x E3 – Hank’s | 18, 18 | 0.8761 | 0.333333 | 2.13166 | -3.90645 | 4.57312 |
| 1x E2 – 1x E3 MB | 21, 18 | 0.319 | 2.059524 | 2.054119 | -2.02604 | 6.145084 |
| 1x E2 – 1x E2 MB | 21, 13 | 0.6521 | 1.021245 | 2.256822 | -3.46748 | 5.509974 |
| 1x E2 – Hank’s | 21, 18 | 0.7642 | 0.618254 | 2.054119 | -3.46731 | 4.703814 |
| 1x E3 MB – 1x E2 MB | 18, 13 | 0.1893 | 3.080769 | 2.32762 | -1.54877 | 7.710313 |
| 1x E3 MB – Hank’s | 18, 18 | 0.2126 | 2.677778 | 2.13166 | -1.56201 | 6.917564 |
| 1x E2 MB – Hank’s | 13, 18 | 0.863 | 0.402991 | 2.32762 | -4.22655 | 5.032535 |
| ***chd7^ncu101/+^*** | | | | | | |
| 1x E3 – 1x E3 MB | 26, 38 | 0.1758 | 2.398785 | 1.764141 | -1.08457 | 5.882143 |
| 1x E3 – 1x E2 MB | 26, 36 | 0.6627 | 0.78914 | 1.805807 | -2.77649 | 4.354768 |
| 1x E3 – 1x E2 | 26, 38 | 0.5491 | 1.059109 | 1.764141 | -2.42425 | 4.542467 |
| 1x E3 – Hank’s | 26, 33 | 0.6291 | 0.879604 | 1.817624 | -2.70936 | 4.468566 |
| 1x E2 – 1x E3 MB | 38, 38 | 0.0311 | 3.457895 | 1.590175 | 0.31804 | 6.597751 |
| 1x E2 – 1x E2 MB | 38, 36 | 0.8692 | 0.269969 | 1.636277 | -2.96092 | 3.500854 |
| 1x E2 – Hank’s | 38, 33 | 0.9135 | 0.179506 | 1.64931 | -3.07711 | 3.436125 |
| 1x E3 MB – 1x E2 MB | 38, 36 | 0.0531 | 3.187926 | 1.636277 | -0.04296 | 6.418811 |
| 1x E3 MB – Hank’s | 38, 33 | **0.0485** | 3.278389 | 1.64931 | 0.02177 | 6.535008 |
| 1x E2 MB – Hank’s | 36, 33 | 0.9575 | 0.090463 | 1.693803 | -3.25401 | 3.434935 |
| ***chd7^ncu101/ncu101^*** | | | | | | |
| 1x E3 – 1x E3 MB | 17, 14 | 0.5037 | 1.60724 | 2.392046 | -3.15902 | 6.373495 |
| 1x E3 – 1x E2 MB | 17, 20 | 0.6649 | 0.931471 | 2.141738 | -3.33604 | 5.198977 |
| 1x E3 – 1x E2 | 17, 11 | 0.6801 | 1.040107 | 2.512252 | -3.96566 | 6.045879 |
| 1x E3 – Hank’s | 17, 18 | 0.3538 | 2.048693 | 2.195727 | -2.32639 | 6.423774 |
| 1x E2 – 1x E3 MB | 11, 14 | 0.8317 | 0.567133 | 2.659761 | -4.73256 | 5.866822 |
| 1x E2 – 1x E2 MB | 11, 20 | 0.9646 | 0.108636 | 2.437107 | -4.7474 | 4.964677 |
| 1x E2 – Hank’s | 11, 18 | 0.686 | 1.008586 | 2.484685 | -3.94226 | 5.95943 |
| 1x E3 MB – 1x E2 MB | 14, 20 | 0.771 | 0.675769 | 2.312998 | -3.93298 | 5.284519 |
| 1x E3 MB – Hank’s | 14, 18 | 0.8523 | 0.441453 | 2.363077 | -4.26708 | 5.149987 |
| 1x E2 MB – Hank’s | 20, 18 | 0.5979 | 1.117222 | 2.109335 | -3.08572 | 5.320164 |
| **Turn Frequency (by media)** | | | | | | |
| **Comparison** | **(n)** | **p-Value** | **Mean Dif** | **Std Error Dif** | **Lower CL** | **Upper CL** |
| **1x E3** | | | | | | |
| *chd7^+/+^* – *chd7^ncu101/+^* | 18, 26 | 0.5115 | 3.535897 | 5.352362 | -7.178 | 14.24981 |
| *chd7^+/+^* – *chd7^ncu101/ncu101^* | 18, 17 | 0.9811 | 0.140196 | 5.903579 | -11.6771 | 11.95749 |
| *chd7^ncu101/+^* – *chd7^ncu101/ncu101^* | 26, 17 | 0.5353 | 3.395701 | 5.44459 | -7.5028 | 14.29423 |
| **1x E3 MB** | | | | | | |
| *chd7^+/+^* – *chd7^ncu101/+^* | 18, 38 | 0.268 | 5.79737 | 5.189855 | -4.56451 | 16.15925 |
| *chd7^+/+^* – *chd7^ncu101/ncu101^* | 18, 14 | 0.3449 | 6.28077 | 6.601797 | -6.90014 | 19.46168 |
| *chd7^ncu101/+^* – *chd7^ncu101/ncu101^* | 38, 14 | **0.0421** | 12.07814 | 5.827883 | 0.44239 | 23.71388 |
| **1x E2** | | | | | | |
| *chd7^+/+^* – *chd7^ncu101/+^* | 21, 38 | 0.2099 | 6.34699 | 5.013997 | -3.66099 | 16.35497 |
| *chd7^+/+^* – *chd7^ncu101/ncu101^* | 21, 11 | 0.3248 | 6.80779 | 6.86323 | -6.89127 | 20.50686 |
| *chd7^ncu101/+^* – *chd7^ncu101/ncu101^* | 38, 11 | **0.041** | 13.15478 | 6.313489 | 0.55301 | 25.75656 |
| **1x E2 MB** | | | | | | |
| *chd7^+/+^* – *chd7^ncu101/+^* | 13, 36 | 0.384 | 5.0905 | 5.806713 | -6.50975 | 16.69074 |
| *chd7^+/+^* – *chd7^ncu101/ncu101^* | 13, 20 | **0.033** | 13.82462 | 6.343996 | 1.15103 | 26.4982 |
| *chd7^ncu101/+^* – *chd7^ncu101/ncu101^* | 36, 20 | 0.0866 | 8.73412 | 5.01805 | -1.29059 | 18.75883 |
| **Hank’s** | | | | | | |
| *chd7^+/+^* – *chd7^ncu101/+^* | 18, 33 | 0.7942 | 1.394949 | 5.32582 | -9.23839 | 12.02829 |
| *chd7^+/+^* – *chd7^ncu101/ncu101^* | 18, 18 | 0.5872 | 3.305556 | 6.058615 | -8.79086 | 15.40197 |
| *chd7^ncu101/+^* – *chd7^ncu101/ncu101^* | 33, 18 | 0.3807 | 4.700505 | 5.32582 | -5.93284 | 15.33385 |
| **Turn Frequency (by genotype)** | | | | | | |
| **Comparison** | **(n)** | **p-Value** | **Mean Dif** | **Std Error Dif** | **Lower CL** | **Upper CL** |
| ***chd7^+/+^*** | | | | | | |
| 1x E3 – 1x E3 MB | 18, 18 | 0.431 | 4.93333 | 6.234543 | -7.46692 | 17.33359 |
| 1x E3 – 1x E2 MB | 18, 13 | **0.0058** | 19.26795 | 6.807675 | 5.72776 | 32.80814 |
| 1x E3 – 1x E2 | 18, 21 | 0.6718 | 2.55476 | 6.007756 | -9.39442 | 14.50395 |
| 1x E3 – Hank’s | 18, 18 | 0.0704 | 11.42778 | 6.234543 | -0.97248 | 23.82803 |
| 1x E2 – 1x E3 MB | 21, 18 | 0.6932 | 2.37857 | 6.007756 | -9.57061 | 14.32776 |
| 1x E2 – 1x E2 MB | 21, 13 | **0.0132** | 16.71319 | 6.600609 | 3.58484 | 29.84153 |
| 1x E2 – Hank’s | 21, 18 | 0.1435 | 8.87302 | 6.007756 | -3.07617 | 20.8222 |
| 1x E3 MB – 1x E2 MB | 18, 13 | **0.0383** | 14.33462 | 6.807675 | 0.79442 | 27.87481 |
| 1x E3 MB – Hank’s | 18, 18 | 0.3006 | 6.49444 | 6.234543 | -5.90581 | 18.8947 |
| 1x E2 MB – Hank’s | 13, 18 | 0.2528 | 7.84017 | 6.807675 | -5.70002 | 21.38036 |
| ***chd7^ncu101/+^*** | | | | | | |
| 1x E3 – 1x E3 MB | 26, 38 | **0.0016** | 14.2666 | 4.444851 | 5.49009 | 23.04311 |
| 1x E3 – 1x E2 MB | 26, 36 | **0.0001** | 17.71335 | 4.54983 | 8.72955 | 26.69715 |
| 1x E3 – 1x E2 | 26, 38 | **0.0058** | 12.43765 | 4.444851 | 3.66114 | 21.21416 |
| 1x E3 – Hank’s | 26, 33 | **0.0005** | 16.35862 | 4.579606 | 7.31604 | 25.40121 |
| 1x E2 – 1x E3 MB | 38, 38 | 0.6486 | 1.82895 | 4.006535 | -6.08209 | 9.73999 |
| 1x E2 – 1x E2 MB | 38, 36 | 0.2025 | 5.2757 | 4.12269 | -2.8647 | 13.41609 |
| 1x E2 – Hank’s | 38, 33 | 0.3468 | 3.92097 | 4.155527 | -4.28426 | 12.1262 |
| 1x E3 MB – 1x E2 MB | 38, 36 | 0.4043 | 3.44675 | 4.12269 | -4.69365 | 11.58714 |
| 1x E3 MB – Hank’s | 38, 33 | 0.6153 | 2.09203 | 4.155527 | -6.11321 | 10.29726 |
| 1x E2 MB – Hank’s | 36, 33 | 0.7513 | 1.35472 | 4.267629 | -7.07186 | 9.78131 |
| ***chd7^ncu101/ncu101^*** | | | | | | |
| 1x E3 – 1x E3 MB | 17, 14 | 0.8596 | 1.20724 | 6.800608 | -12.3433 | 14.75775 |
| 1x E3 – 1x E2 MB | 17, 20 | 0.3621 | 5.58353 | 6.088982 | -6.549 | 17.71609 |
| 1x E3 – 1x E2 | 17, 11 | 0.5665 | 4.11283 | 7.142356 | -10.1186 | 18.34429 |
| 1x E3 – Hank’s | 17, 18 | 0.1897 | 8.26242 | 6.242472 | -4.176 | 20.70081 |
| 1x E2 – 1x E3 MB | 11, 14 | 0.7019 | 2.90559 | 7.561725 | -12.1615 | 17.97266 |
| 1x E2 – 1x E2 MB | 11, 20 | 0.1659 | 9.69636 | 6.928716 | -4.1094 | 23.50213 |
| 1x E2 – Hank’s | 11, 18 | 0.0839 | 12.37525 | 7.063983 | -1.7 | 26.45055 |
| 1x E3 MB – 1x E2 MB | 14, 20 | 0.3051 | 6.79077 | 6.575875 | -6.3119 | 19.89349 |
| 1x E3 MB – Hank’s | 14, 18 | 0.1629 | 9.46966 | 6.71825 | -3.9167 | 22.85606 |
| 1x E2 MB – Hank’s | 20, 18 | 0.6564 | 2.67889 | 5.996859 | -9.2701 | 14.62789 |

**Supplemental Table 4.** **Multiple comparisons of *chd7* mutants within and between media types.** Multiple comparisons of *chd7^+/+^, chd7^ncu101/+^,* and *chd7^ncu101/ncu101^*  in 1x E3, 1x E3 MB, 1x E2, 1x E2 MB, of Hank’s and their reported values for the acoustic startle response (normalized SLC and LLC Index), and general locomotor behaviors (total distance, swim and turn frequency) with statistically significant p-values in bold (α=0.05, ANOVA with student’s t each pair test for multiple comparisons).
