# Supplementary material for: Effects of 4 Testing Arena Sizes and 11 Types of Embryo Media on Sensorimotor Behaviors in Wild-Type and chd7 Mutant Zebrafish Larvae
Source: Zebrafish. 2024 Feb 14;21(1):1–14. doi: 10.1089/zeb.2023.0052 (PMC10902501; doi:10.1089/zeb.2023.0052)
Supplement: Supplemental data [file Suppl_FigS3.docx]

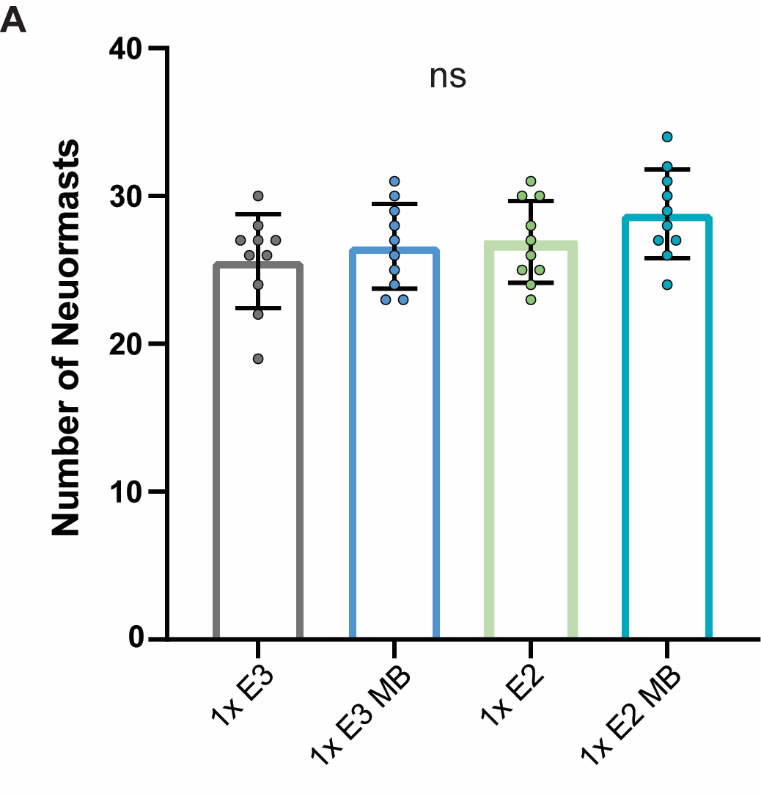


**Figure S3. Neuromast counts in larvae reared in media with or without methylene blue. (A)** Quantification of total neuromast numbers including head and trunk (1x E3: n= 10; 1x E3 MB: n=10; 1x E2: n=10; 1x E2 MB: n=10, (mean ± SD, Ordinary one-way ANOVA with Tukey’s multiple comparisons).
